# Long-term groundwater protection efficiency of different types of sanitary landfills: Model description

**DOI:** 10.1016/j.mex.2020.100810

**Published:** 2020-02-21

**Authors:** Igor Madon, Darko Drev, Jakob Likar

**Affiliations:** a Komunalno stanovanjska družba, Goriška 23B, 5270 Ajdovščina, Slovenia; b Faculty of Civil Engineering and Geodesy, University of Ljubljana, Slovenia; c Faculty of Natural Sciences and Engineering, University of Ljubljana, Slovenia

**Keywords:** Risk assessment, Sanitary landfills, Groundwater protection, Pollutant emissions, Leachate leakage

## Abstract

A new risk assessment method intended for comparing long-term environmental performance of different types of sanitary landfills was customized. Processes occurring within the hydrogeological environment were excluded from modeling, because they can be addressed separately. Only parameters directly related to leachate composition at the bottom of the landfill and leachate losses into the subsoil after landfill closure which can be reliably determined by evaluating already available information from the scientific literature were considered as necessary inputs for quantitative modeling. Once the simulated outcomes for a primary output ‘’fugitive emissions of a reference pollutant into the subsoil’’ are acquired, more complex outputs can be derived, too. Commercially available risk assessment software which operates within an Excel environment was used to fulfill the task.

• Uncertainty of data as well as heterogeneity and complexity of landfill systems was considered by attributing the selected input parameters with adequate probability density functions
• Probability density functions attributed to the inputs differ considerably between the antagonistic landfill types
• Risk assessments related outputs were defined as probabilities that an aquifer would be polluted due to landfill derived emissions into the subsoil

## Graphical abstract

**Figure:**
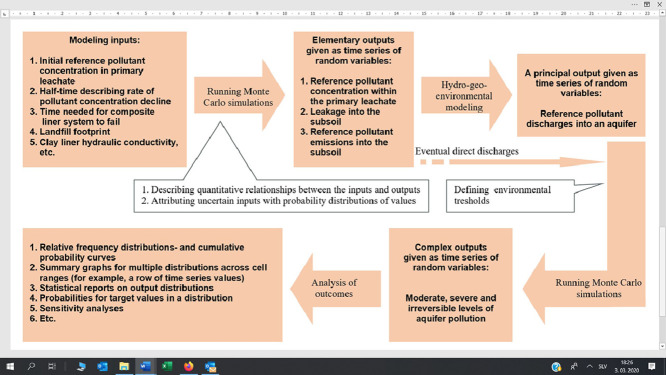

**Specification Table**

**Table:** 

Subject Area	Environmental Science
More specific subject area	Waste disposal Groundwater protection
Method name	Long-term risk assessment model for sanitary landfills
Name and reference of original method	LandSim 2.5 (Environment agency, 2004) Life cycle assessment (e.g., Turner et al., 2017)
Resource availability	• https://doi.org/10.1016/j.wasman.2019.07.001 • https://doi.org/10.1016/j.dib.2019.104488

## Method details

### Background

The article is closely related to a research article ‘’Long-term risk assessments comparing environmental performance of different types of sanitary landfills” [1] and Data in Brief article “Long-term groundwater protection efficiency of different types of sanitary landfills: data description” [2].

Accurate modeling of leachate derived pollutant emissions from sanitary landfills into the surrounding hydrogeological environment is an extremely difficult task to be accomplished as the number of factors which affect contaminant migration is too high and need to be limited when making a model [3]. Probabilistic methodology is usually applied to landfill risk assessment models because it allows quantification of ubiquitous uncertainty when specifying hydro-geological environment, landfill leachate chemistry and/or performance of landfill lining systems. The most known software in use today is LandSim 2.5 [4], which was primarily designed to calculate environmental permit- related outputs such as leachate head at the bottom of landfills and concentration of pollutants at the correspondent compliance points. However, deterministic models are sometimes used, too. For example, Turner et al. [5] developed a specific model for evaluation of different landfill aftercare strategies based on “Life Cycle Assessment” (LCA) approach.

None of the already known models seemed to be adequate for performing long-term risk assessments comparing groundwater protection effectiveness of landfills of different types, which was the objective of the related research article [1]. Consequently, a specific risk-assessment model was designed for the purpose, which is presented in this article.

## Types of sanitary landfills

Suitable categorization of sanitary landfills into different types is very important in order to understand the concept upon which the presented risk assessment model was developed.

Sanitary landfills are facilities for disposal of untreated, mixed municipal solid waste (MSW) as a principal waste stream. Landfills for disposal of mechanically-biologically pre-treated residual MSW or for disposal of waste-to-energy derived bottom ash do not comply with such definition.

Sanitary landfills can be divided into opposite groups from many different aspects, e.g.

• “modern’’ vs. “old ’’, “higlhy- engineered-” vs. “’poorly-engineered’’, “high-cost-” vs. “low-cost-”, “uncontained’’ vs. “contained’’ facilities
• “dry-type’’ vs. “wet-type’’ facilities (the later category includes bioreactor landfills)
• “anaerobic’’ vs. “semi-aerobic’’ landfills (aerobic also exist)
• “above-ground’’ vs. “pit and mound’’ facilities (the later category includes “below-ground’’ landfills)
• “active’’ vs. “closed’’, “non-compartmentalized’’ vs. “compartmentalized’’ landfills, etc.

Borderlines which separate sanitary landfills into opposite categories appear to be vague. For example, landfills which were considered to be modern in 1980s may not be considered to be modern from a present-day perspective. A particular landfill can be at the same time poorly-engineered, wet-type, semi-aerobic, above-ground, compartmentalized, etc.

When separating landfills into antagonistic types specifically to quantitatively compare their long-term groundwater-protection efficiency after landfill closure, the most important characteristics to be scrutinized appear to be -

• the design of bottom liner- and capping systems
• landfilling methods which are/were applied and
• approaches eventually employed to stabilize the buried waste

Many types of sanitary landfills can be defined based on these criteria, however, in the related research article [1] only four categories of landfills were distinguished. Categorization was performed in a way that

• landfills which broadly demonstrate similar long-term environmental characteristics were grouped together to form one landfill type
• high-cost and low-cost facilities were classified as separate types

Two high-cost- and two low-cost landfill types were predisposed. Landfills bottom-lined with composite liner systems were automatically considered to be “modern” in the research article. Closed modern landfills covered only with soil located in humid climate environments were considered to be of a wet-type, since leachate generation can sometimes amount up to 60% of annual precipitation [6]. Such landfills generally stabilize fast when compared to thoroughly sealed landfills where composite liners were implemented for capping (the later were considered to be modern landfills of a dry-type).

Low-cost landfill types were also divided into two broad categories: (1) uncontained dumpsites and (2) contained, clay-only lined waste deposits. Each of these two categories includes subtypes which affect the environment extremely differently. For instance, dumpsites constructed as above ground waste piles generally emit much less pollutants into the subsoil than dumpsites located in abandoned pits or natural depressions. It would be senseless to group them together as a common type in order to perform comparative risk assessments. Consequently, low-cost landfill types were represented only by the subtypes which perform the best from the long-term groundwater vulnerability point of view (i.e., represented by those which on average stabilize the fastest and emit the smallest amounts of pollutants into the subsoil). These sub-types are represented by the “above-ground semiaerobic dumpsite’’ (subgroup appertaining to the uncontained landfills category) and “high-permeability landraise’’ (subgroup appertaining to the contained, clay-only lined landfills). Other subtypes of low-cost landfills were not considered since it is obvious that they behave environmentally much worse than the two mentioned above within their categories. However, it has to be taken in mind that large number of landfills operating today in low-income developing countries belong to environmentally “bad subtypes’’, such as

• below-ground and “pit and mound’’ dumpsites
• below-ground and “pit and mound’’, clay-only lined anaerobic waste deposits
• above-ground, anaerobic, clay-only lined waste deposits (these landfills may look similar to high-permeability landraises, however, they appear to be inherently of a “non-flushing-’’ instead of a “flushing’’ type and conditions within their interior to be anaerobic rather than semiaerobic, which is due to higher in-place densities of the buried waste, impermeable final cover design, etc.)

Characterization of the four types/subtypes which were compared [1] is outlined below:

1. Dry-type modern landfills: (a) composite bottom liner- and composite cover systems are installed; (b) highly engineered systems for leachate and landfill gas capture, collection, and treatment are provided; (c) buried waste is heavily compacted; (d) leachate recirculation is not implemented.
2. Wet-type modern landfills: (a) composite bottom liner and mineral or composite cover systems are installed; (b) highly engineered systems for leachate and landfill gas capture, extraction, collection, and treatment are provided; (c) buried waste is heavily compacted; (d) water recirculation and other waste stabilization activities start after landfill closure, which may include controlled air injection; (e) landfills that were not capped with composite liners and are located in humid regions are also considered to be of a wet-type even if not practicing leachate circulation
3. Above-ground semiaerobic dumpsites: (a) erected as relatively narrow above-ground waste piles, (b) contain no liner at the bottom of the landfill, (c) has buried waste that is loosely compacted, (d) minimal sanitary covering is provided during the time the dumpsite receives waste; waste soils and/or construction and demolition (C&D) waste are used for this purpose, (e) some soil is provided as a final cover.
4. High-permeability landraises (HPL's): (a) above-ground waste pile is designed in a way that passive aeration of the landfill interior is provided; (b) clayey barrier and low-cost leachate drainage system are provided at the bottom of the landfill; (c) buried waste is loosely compacted using only a bulldozer; (d) multi-branched recirculation system for in-situ treatment of leachate and other facility-derived wastewaters is installed, which includes a landfill body flushing component activated immediately after landfill closure.

Modern dry- and wet-type landfills could be divided into smaller groups (e.g., by introducing “state-of-the-art waste disposal facilities” as separate dry- and wet- branches of modern landfills), however, older and newer modern landfills are not antagonistic to one another but rather represent continuums of conceptually equal landfills as described in Section ``*Approaches used to calculate leakings when referring to the companion research article”*.

### Reasons for introducing “high-permeability landraise” (HPL) as a specific subtype of a contained, low-cost landfill

In 1999, so called “Landfill Directive’’ [7] was issued in the EU. Actively operating sanitary landfills were already gradually disappearing in Germany and other highly industrialized EU countries. These facilities were progressively supplanted with landfills for disposal of mechanically-biologically pre-treated residual MSW or landfills for disposal of MSW-to-energy derived bottom ash. EU Landfill Directive has not addressed the issue of using alternative, more sustainable sanitary landfilling concepts for bridging transitional time until integrative waste management (WM) systems would be established in other parts of the EU, too [8]. Although it was already known that disposal strategy involving waste encapsulation does not bring the buried waste closer to final storage quality and implies acceptance of an indefinite responsibility for a potential environmental risk on behalf of future generations, modern dry type landfill remained to be considered as a reference type of facility in many national regulations within the EU. On the other hand, Final Report for the Swedish EPA in 2000 [8] explicitly recommended new landfill concepts to be developed and implemented rather than using old ones, such as avoiding below ground landfilling, identifying critical components in defining “final storage quality”, employing strategies to minimize short and long term impacts on the environment, providing passive environmental protection systems in the final stage of landfill life, developing methods and technologies to ensure uniform distribution of water across the volume of the landfill, investigating possibilities for accelerated flushing of the landfill interior, etc.. “High permeability landraise” type of landfill [1] was largely developed on basis of these recommendations at the same time seeking to find low-cost waste- disposal solutions [9,10]. This type of landfill seems to be espetially suitable for purposes of flexibly bridging the needed transitional time in low-budget environments where local authorities seek to gradually transform their former dumpsites into safe disposal facilities and further into integrative WM sites.

In the related research article [1], HPL's were represented as a heterogeneous group of landfills in order the performed comparative risk assessments evaluating environmental effectiveness of different landfill types would be based on the same premise. Probability distributions for the inputs required for modeling were selected in a way to consider probable differences which would occur in real- world environment if HPL's were constructed in large numbers. E.g., if such kind of a low-cost facility had to be implemented in some less developed country today, the capacity and waste composition would certainly differ from the one which was involved in performing the research. It is also unlikely the facility would be constructed and operated exactly in a way as intended. Human factors and errors have to be integrated into risk analyses as is inherently the case with other landfill types, too. E.g., looking retrospectively, highly engineered landfills were not supposed to leak when they were designed, but some of them nevertheless leaked soon after they were constructed. Therefore, among other differences, decadic orders of magnitude different hydraulic conductivities “k_sat_” were selected as probable values in order to perform simulations using the proposed model (Fig. 4).

Statistical parameters used to define the presumed log-normal distribution of ‘k_sat_’ (mean = 5 · 10^−10^ m/s, st. deviation = 7.19 · 10^−10^ m/s) may be considered to be too unobjectionable for a low-cost type of landfill. However, hydraulic conductivity of a bottom clayey liner and its thickness are among the most essential parameters defining HPL as a landfill type. In the absence of an adequate clayey barrier such facility can be regarded as a conventional “above ground waste deposit”. Considerable clay liner thickness of 1.1 m–1.5 m which was used in the comparative model (Fig. 4) was meant to be an appropriate measure for preventing contamination in extremely vulnerable hydrogeological settings (the aquifer was assumed to be situated directly underneath the landfill). In most of realistic settings clay liner of 1.0 m is all what is needed to effectively prevent excessive emissions from such a type of facility.

## Description of the applied approach developing the model

### Conception of the method

Logic used in developing the model was driven by the recognition that long-term leachate pollution-potential from sanitary landfills can be quantitatively established if post-closure time-dependent variable “QRP_t_” (reference pollutant annually released into the subsoil) was known.

Just two quantities are needed to derive 'QRP_t_’ according to the proposed model:

1: probability distribution of values for time- dependent variable ‘C_t_’ across the post-closure time period (reference pollutant concentration within the leachate at the bottom of the landfill)

2: probability distribution of values for time- dependent variable 'Q_t_’ across the post-closure time period (annual leachate leakages into the subsoil)

The problem is that these variables already represent quantities on the output side of the model. However, an important point is that the outputs 'Q_t_’ and ‘C_t_’ can be obtained by performing Monte Carlo simulations utilizing relatively small number of input variables which can be convincingly attributed with probability density functions processing already available data and information.

Once the simulated data for time-dependent variable 'QRP_t_’ are known, they can be used to derive other, more complex outputs. The most valuable asset which can be potentially threatened by the landfill appears to be an aquifer utilized as a drinking water supply source. Groundwater threshold values are usually given in terms of pollutant concentrations at compliance- point wells and set according to the requirements imposed by the regulations. However, if the considered aquifer was well explored, these values can also be given directly in terms of threshold discharges of pollutants into the aquifer. Levels of possible landfill-derived pollution can be labelled e.g. as moderate-, severe- and/or “irreversible-”.

If the aquifer was positioned directly underneath the landfill, separated just by a narrow, permeable vadose zone, yearly fugitive emissions into the subsoil would be equal to annual discharges into the aquifer. Separate hydrogeologic transport model would not be required in this case. Operating landfills situated directly above an aquifer are rarely found anywhere in the world today. However, the concept can be adequate for specific modeling purposes, such as for quantitatively comparing long-term groundwater-protection efficiency of different types of sanitary landfills. This was the objective when referring to the companion research- and data- description articles [1,2].

### Identifying suitable modeling inputs

Sanitary landfills around the world dramatically differ among themselves not just from points of view of their capacities and waste composition, but also by their design, mode of operation, waste placement conditions, initial in-place densities of waste, climate in which they are located, etc., to mention just a few. Myriad of combinations exist in regard to how all of these factors may interact between themselves. Large differences exist also among the sanitary landfills appertaining to the very same type/category. Even when dealing with a single landfill and a lot of data is already available, it is still difficult to estimate probability and magnitude of threat the site imposes to adjacent groundwater bodies in the long term. It seems to be impossible to perform quantitative risk assessments in a reasonable way trying to process so many indirectly and/or stochastically related elements, many of which are unknown. Better approach would be to simplify the complex system in order to exploit simplicities without deconstructing intricate complexities.

According to the applied approach, vast number of interrelated factors which influence long-term groundwater- protection performance of sanitary landfills can be reduced to few general determinants: 1) Overall hydrogeological setting; 2) Landfill footprint; 3) Landfill characteristics.

**Fig. 1:**
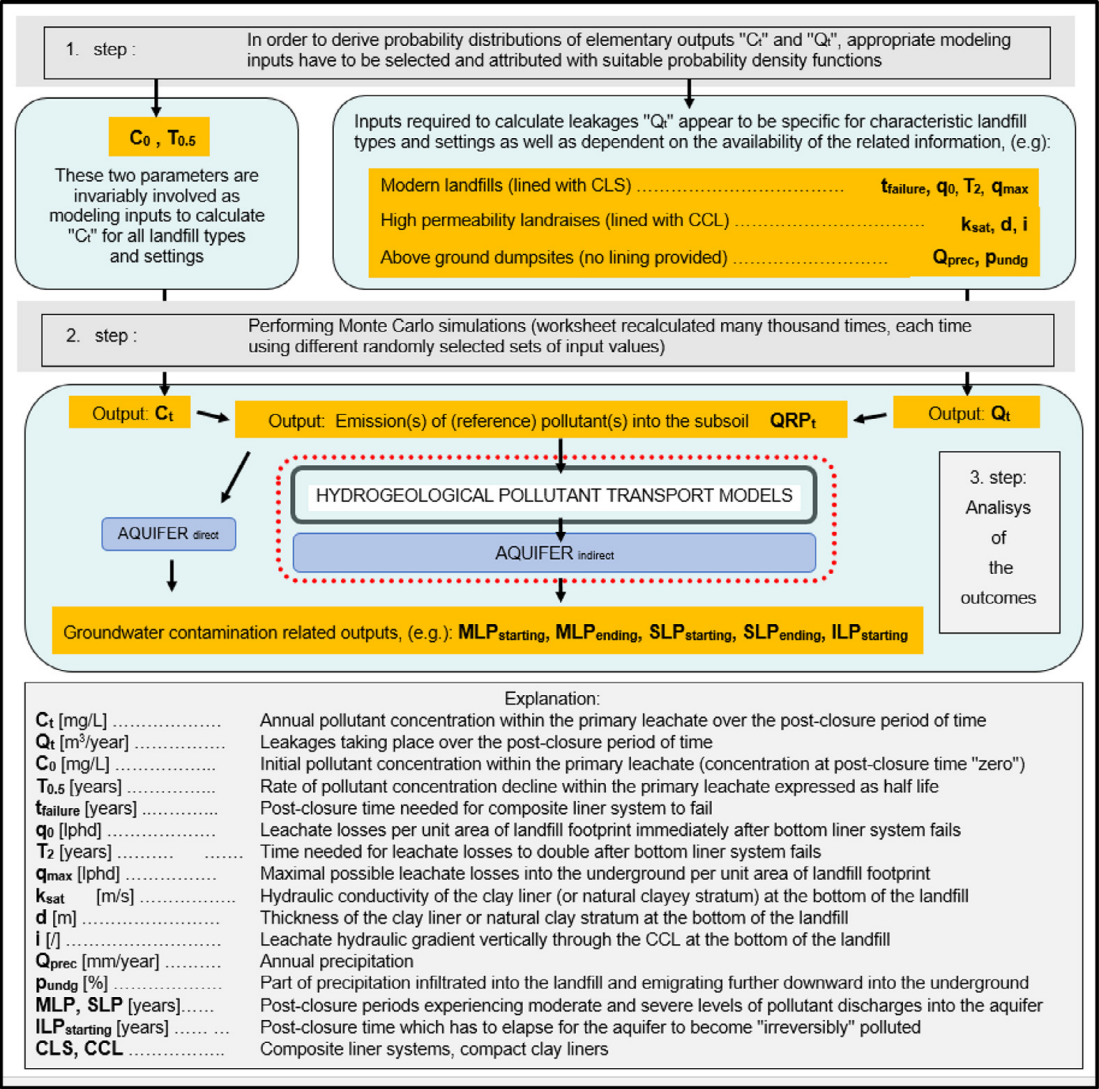
Flowchart of the applied method concept.
    iii. Hydraulic systems at the bottom of clay-only lined landfills are considered to be deterministic. Long-term leakage rates are calculated using the Darcy law, nevertheless, values of the required inputs 'k_sat_’, 'd' and ‘i’ (Fig. 1) usually appear to be uncertain, therefore, they have to be quantitatively characterized as random variables, making this part of the model to be probabilistic, too. When performing general risk assessments studying landfill types as groups, the spread of possible values for these variables is inherently larger in order to consider for diversity of landfills apparteining to particular landfill types.
    iv. Data are usually very deficient when evaluating environmental impacts from uncontained landfills (dumpsites). Leachate infiltration into the subsoil is prevalently dependent on local hydrogeologic and climate conditions. If attributed with appropriate distributions of values and their probabilities, ‘Q_precip_’ and ‘p_undg_’ (Fig. 1) appear to be practical, reliable input parameters to be applied to estimate annual leachate discharges from dumpsites into the immediate underground.
  b. groundwater- contamination- related pollution- potential accumulated at the site during the landfill pre-closure phases:
    i. Usually, only a very small part of the overall pollution potential which was accumulated at the site before the landfill was closed ends up in the form of fugitive emissions of aqueous pollutants into the subsoil after the landfill was closed. Major part of the accumulated pollution potential rather ends up in the form of treated leachate and treated landfill gasses (which is something to be expected when referring to contained types of landfills, at least during the first 30 years after landfill closure) or as direct pollution fluxes into the surface waters and into the atmosphere (which are common circumstances when referring to uncontained landfills). When evaluating groundwater protection performance of landfills of different types these other fluxes are in principle not important as long as they are not needed for calculation of emissions of pollutants into the subsoil. Landfill's potential to generate specific amounts of aqueous pollutants during the post-closure time is therefore not equal to groundwater- contamination- related pollution- potential according to the applied concept.
    ii. Only data regarding concentration of reference pollutants within the leachate at the bottom of the landfill and the related length of post-closure time that such potentially harmful leachate exists there appears to be indispensable information to evaluate possible pollutant discharges into the subsoil. Pollution potential is generally the greatest during the time a landfill ceases to receive new waste and the landfill is closed. According to the applied approach, this initial pollution potential is expressed by introducing the input parameter ‘’initial reference pollutant concentration ’C_0_’’’. ’C_0_’ can be expressed as a discrete value only in cases when evaluating emissions from factual, already closed landfills where the parameter was actually measured. Otherwise, the input is considered to be a random variable quantified by a probability density function derived on basis of processing secondary data from scientific literature and other sources.
  c. expected rate of pollution-potential decline during the landfill post-closure phases:
    i. After closure, reference pollutant concentration values within the primary leachate at the bottom of the landfill generally start to drop. The faster this process proceeds, the smaller
      1 the probability for groundwater to become contaminated, and/or
      2 the magnitude of eventual groundwater contamination, and/or
      3 the length of time the threat is present at the site and/or the pollutants can be emitted into the subsoil
    ii. Annual average concentration of reference pollutant(s) within the leachate at the bottom of the landfill are good indicators describing the acquired level of landfill stabilization and its remaining potential to pollute groundwater in the future. When reference pollutant concentration becomes so low that primary leachate cannot cause harm to the adjacent subterranean environment any more, the related landfill pollution potential can be considered to be exhausted and the particular landfill to be stabilized.
    iii. The rate of pollutant concentration decline at the bottom of the landfill is considered to be approximately of the pseudo-first order rate, therefore expressed as half-life period ‘T_0.5_’ (time needed for reference pollutant concentration to be reduced by 50%). This input has to be always considered uncertain for modeling purposes (therefore characterized by a probability density function) even if the value was acquired by performing measurements on a factual waste disposal site which was already closed over a long period of time.

1) Overall hydrogeological setting is meant to be quantitatively evaluated only after the results regarding pollutant emissions into the immediate subsoil are already attained. The task regarding transport of pollutants and their fate in the subterranean environment is therefore meant to be tackled separately, using one of the already existing hydro-geo-environmental models.
2) Landfill footprint is one of the few input parameters which are already known or can be determinedly assumed, therefore quantified with a discrete value.
3) Ample amount of information which usually exists describing characteristics of a particular landfill (such as landfill-type, -design, -capacity, waste composition, quality of landfill construction, etc.) is meant to be filtered out in a way to find answers to essential questions regarding the –
  a. reliability of implemented bottom liner systems:
    i. Leakages from landfills are mostly related to hydraulic characteristics of natural and/or artificial barriers situated at their bottom and to different deterioration processes gradually affecting performance of composite leachate containment and conveyance systems eventually installed there.
    ii. Long-term leachate losses into the subsoil are usually considered to be a stochastic phenomenon when referring to modern landfill types (which are inherently considered to be bottom lined with composite liner systems). Set of random variables which were employed as model inputs in order to perform simulations in the companion research article [1] consisted from 't_failure_’, 'q_0_’, ‘T_2’_and 'q_max_’ (Fig. 1). Other approaches can be implemented, though, which is touched in Section ``*Leakage through composite liner systems and the related affecting factors”*.

Flowchart demonstrating the applied modeling concept is presented in Fig. 1.

### Determination of inputs–outputs relationships

As mentioned in Section ``*Conception of the method”*, there are just two decisive quantities which are ultimately important to perform the necessary calculations to evaluate long-term pollutant emissions into the subsoil 'QRP_t_’: 1) ‘’primary leachate losses into the subsoil’’ and 2) ‘’concentration of pollutants within the leachate at the bottom of the landfill’’. Both quantities generally change over time after landfill closure (Q_t_, C_t_).

Since many of the modeling inputs are inherently quantified through probability density functions, the derived outputs, too, can be nothing but quantified with probability distributions of possible outcomes. The task can be comfortably accomplished utilizing appropriate softwear tool which uses established mathematical algorithms (such as Monte Carlo algorithm) to select random values in order to perform simulations in which many recalculations are required. When using @Risk [11], which is an add-in to Microsoft Excel, uncertain inputs are conveniently entered as probability density functions in cell formulas. The program is mostly used in economic sciences, however, it is frequently applied in environmental sciences, too. It allows extraction of meaningful statistics for the desired outputs.

#### Calculation of long-term pollutant concentration decline in primary leachate

Long-term decline in the concentration of primary leachate pollutants after landfill closure is satisfactorily described by pseudo-first-order rate kinetics: C_t_ = C_0_ · *e*^−kt^ [12]. Values for the constant “k” are derived from the correspondent half-lives: *k* = ln2/T_1/2_. Therefore, concentration of pollutants within the primary leachate ‘C_t_’ can be calculated if probability distributions of random variables ‘C_0’_(initial concentration of the pollutant immediately after landfill closure) and ‘T_0.5_’ (half-life period characterizing rate of pollutant concentration decline) are entered into the model.

As outlined in Section `**`*Identifying suitable modeling inputs”***, ‘C_0’_value appears to be one of the outcomes resulting from all those interconnected processes convoluting at a particular disposal site during the pre-closure phases. On the other hand, ‘T_0.5_’ is related to the nature and intensity of biological, chemical and physical processes occuring within the landfill during the period of time after the facility was closed. ‘C_0’_and ‘T_0.5_’ values cannot be calculated by quantitatively considering all of the above mentioned processes many of which are unknown and/or stochastic in nature. However, these quantities can be measured (if dealing with the factual, already closed facilities) or can be reliably estimated by landfill experts on basis of general knowledge they have in the field, namely, these values tend to cluster together around the averages which are characteristic for particular landfill types. Therefore, part of the work needs to be done qualitatively using sound professional judgment according to the proposed methodology. Such approach is common in other fields, too, when dealing with uncertain and stochastic inputs [11,13].

When loosely defined groups of landfills are to be compared, which was the objective of the related research article [1], probability distributions for the inputs ‘C_0’_and ‘T_0.5_’ can be nothing but approximate and spread out. Approaches and techniques which were used to derive their averages and standard deviations are presented in Section ``*Derivation of probability distributions for the inputs ‘C_0_’ and ‘T_0.5_’”*.

#### Calculation of long-term leachate losses into the subsoil

As opposed to the parameter ‘C_t_’, calculation of leachate losses ’Q_t_’ into the underground cannot be applied in a common manner when referring to different landfill types, landfill designs, etc., because the related hydraulical settings appear to be inherently diverse, as contemplated below:

1) Fugitive flow of leachate needs to be evaluated at an annual level for modeling purposes, therefore, quasy steady-state flow situations occuring at the bottom of already closed clay-lined landfills can be hardly assessed in the same way as transient flow situations occuring sporadically at the bottom of inadequately capped above ground dump sites.
2) While hydraulic properties of compact clay liners (CCL's) are not expected to change significantly through the decades (espetially not for the worse), characteristics of composite liner systems (CLS's) are probabilistically expected to change [14,15]. Even CCL's which are constituent parts of CLS's do not behave in the same manner as CCL's acting as sole elements of bottom- liner sealing systems, at least not probabilistically (the later are inherently continually water-saturated, consequently, clay minerals remain in a maximally swollen condition all the time, which can not be claimed for CCL's that reside in the vadose zone below the synthetic geomembrane). While transport of pollutants through CCL's is dependent on hydraulic and chemical concentration gradients and permeability and diffusivity of the related compacted clays [15,16] leakages through CLS's appear to be dependent on a much larger number of known and unknown factors interacting between them in the short- as well as in the long-term.
3) Darcy's law describes flow of a fluid through a porous medium such as CCL. It is not intended to be used for calculating flow through synthetic geomembranes (although permeability through composite liner systems is sometimes given by an all-encompassing hydraulic coefficient value – e.g. [5]). Vice versa, when comparing environmental performance of different types of landfills, it would be factitious to ignore the fact that hydraulic conductivity is an essential property of clayey barriers just to show that equal probabilistic method was used to calculate long-term leakages considering both, clay-only-lined landfills and landfills equipped with composite liner systems.
4) Based on reasonings specifyed above, relations between leakages and input variables required to calculate these leakages can be deterministic in some settings (Darcy law can be applied in situations where hydraulic conductivity and thickness of the clayey barrier at the bottom of the landfill are approximately known) while stochastic in other settings (e.g., already constructed failure probability curves based on documented environmental performance of modern-type landfills can be used to evaluate timing and the related probabilities of composite liner system failure).

##### Leakage through composite liner systems and the related affecting factors

Reliability of composite liner systems and consequently of their failures depends on several events, each characterized by an actual probability. Analysis usually entails knowledge of failure probability of the individual elements (subsystems) and combines them with an appropriate probabilistic analysis to define the reliability of a more complex system [17,18]. A Fault Tree is widely used to assess the failure of a “technological system” [12].

Estimated landfill leakages through the geomembrane (GM) are often calculated using Bernoulli or Giroud equations if the hole size and frequency are known or presumed [19]. Leak frequency and size statistics are normally generated from the results of geoelectric leak location methods [20]. Average hole size and frequency contributing to leakage depends heavily on the skill of the liner installer and the skill of the construction-quality-assurance (CQA) agency. GM/CCL composites are better in preventing leakage than GM's or CCL's alone if GM is in direct contact with CCL and temperatures at the landfill bottom are smaller than 40 °C in the long term [15]. For example, calculated leakage when considering 2.5 small holes/ha and 30 cm water head appears to be just 1 lphd. However, if that holes appear to be located within the GM system of interconnected wrinkles (waves) of length 200 m/ha (such circumstances are sometimes observed when performing liner integrity surveys before waste disposal operations begin), the calculated leakages rise to 120–170 lphd [15].

Mathematical models for advection-dispersion of pollutants through layered bottom liners (such as through a composite geosynthetic clay liner/ attenuation layer system) have usually been solved numerically or using analytical solutions [3].

According to the analysis which quantitatively scrutinized leakage performance of GM/CCL systems [21] and the importance of particular components involved by altering

• hydraulic head (from 0.3 m to 10 m)
• GM thickness (from 0.5 mm to 2.5 mm)
• number of GM defects (from 2 to 200 holes/ha)
• CCL thickness (from 0.5 m to 3 m) and
• hydraulic conductivity of CCL (from 1 × 10^−8^ m/s to 1 × 10^−10^ m/s),

CCL thickness appears to have the greatest impact on CLS performance. Other parameters being constant, numerically calculated critical time for reaching critical concentration of Cd^2+^ on the bottom part of the CCL was

• 11 to 80 years (by altering water head)
• 75 to 83 years (by altering GM thickness)
• 27 to 153 years (by altering number of holes/ha), however, as much as
• 44 to >1000 years (by altering CCL thickness) and
• 33 to 147 years (by altering k_sat_ of the CCL).

Modern landfills for disposal of untreated MSW are generally very anaerobic. Also, footprints of hydraulically separate compartments normally appear to be relatively small in order to be filled with waste as rapidly as possible, preventing excessive rainwater to enter the buried waste. Consequently, even if the amount of generated leachate is small, the generated leachate can be very loaded with both, organic and inorganic substances eventually causing heavy precipitation of calcite, iron colloids and humic material on locations whereever oxidation/reduction potential (ORP) suddenly increases. Subsequently, such conditions often result in cloggings and incrustations of leachate drainage and collection systems [22], [23], [24] and leachate mounds with excessive water heads can be induced potentially increasing the intensity of eventual leakings. Many other factors and mechanisms can provoke leakage increases in the short- as well as in the long term [15].

Bioreactor operation is not recommended by geotechnical experts because high leachate temperatures can induce water vapor movement and dessication cracking in clayey liners lying underneath geomembranes, espetially if liner thickness was small. Estimated service life of GM is heavily dependent on landfill liner temperature-time hystory, too, which can range roughly from 20 to 3300 years in real life landfills [25].

All-encompassing way to assess performance of modern landfills and their CLS's is by obtaining data from monitoring wells lying downstream of as large number of modern-built landfills as possible. All the factors and interactions between them which could have caused the failure are in this manner factored in, including the fact that GM and CCL for some reason did not prevent the leak. However, groundwater- protection effectiveness cannot be acquired in this way when referring to the contemporary, state-of-the-art landfills, because too little or no post-closure time has expired so far in order to evaluate performance of this particular sub-group of modern landfills.

##### Approaches used to calculate leakings when referring to the companion research article

Approaches which were used to calculate long-term leakages ‘Q_t_’ are described below:

###### Above-ground dump sites

Annual quantity of water infiltrating into the waste pile and further emigrating downwards into the underground was estimated as a percentage of precipitation based on the presumed hydrologic and hydrogeologic characteristics of the site and its surroundings.

$$Q_{t}\;=\;Q_{\text{precip}}\cdot A\cdot p_{\text{undg}}$$

Q_precip_: annual precipitation [mm];
A: landfill footprint [m^2^];
p_undg_: part of annual precipitation which is infiltrated into the landfill generating landfill leachate, but only that portion which percolates further down into the subsoil [%]

###### High-permeability landraises (HPL's)

Leachate flow through the saturated clayey barrier underneath the landfill was calculated according to the Darcy's law. Quantity of leachate released into the subsoil was considered to be zero until the time leachate pollutants penetrate the liner and break through on the other side of the liner.

$$Q_{t}\;=\;0,\;\;\text{if}\;t\;<\;t_{\text{breakthr}};\;Q_{t}\;=\;\;k_{\text{sat}}\cdot A\cdot i,\;\text{if}\;t\;>\;t_{\text{breakthr}};\;\;t_{\text{breakthr}}\;\;=\;\;d/\left(k_{\text{sat}}\cdot i\right)$$

d: clayey liner thickness [m];
i: hydraulic gradient [/];
k_sat_: hydraulic conductivity coefficient [m/s];
t_breakthr_: post-closure time which has to pass for pollutant to penetrate the clay liner [years]

Probability density functions for parameters ‘k_sat_’ and ‘d’ were selected according to the characteristics which define HPL as a landfill type.    

###### Modern landfill types

Leachate losses into the subsoil were considered to be non-existent until the post-closure time when bottom liner system fails. General process which leads to landfill liner system failure was thought-out to be inherently stochastic. Probability distribution of values for the input ‘t_failure_’ (post-closure time which has to expire for bottom liner system to fail, i.e., for leakages to begin) can be derived in different ways, as presented in Section ``*Leakage through composite liner systems and the related affecting factors”*. Approach which was applied in the companion article [1] was to utilize already obtained ’failure probability curve’ based on monitoring data derived from North Italian wells positioned downgradient of landfills bottom lined with CLS's [12]. Since the number of monitored landfills was relatively large, the related failure probability curve which was constructed could be considered to be quite representative for landfills lined with composite liner systems during the 1980s and 90 s.

Initial leakage flow rates into the underground per unit area of landfill footprint ‘q_0_’ after the liner fails could only be very low for modern landfills. Exact measurements could have been historically performed only on real and pilot-scale landfills equipped with double bottom lined systems [14,^26^,27]. Already measured values mainly fell within the 0.1–10 lphd range, much less into 10–100 lphd range. There were also few cases which fell into (100–1000) lphd and ‘no-leaching-detected’ ranges. The highest measured value was 1410 lphd.

Leakage flow rates are likely to increase gradually in the long term [12], but only until reaching some upperbound limit ‘q_max_’ (maximal possible leachate losses into the subsoil per unit area of landfill footprint). This value (or probability distribution of possible values) can only be given arbitrarily, however, realistically: leakages could be hardly larger than they would be if compact clay liner was a sole element of composite liner system. The largest measured value for ‘q_0_’ which was already measured on landfills equipped with double bottom liner systems [27] suggests such proposition to be reliable. Rate of increase in leachate flow rates can be conveniently described by first-order rate kinetics (e.g., values can be given by the parameter “time needed for leachate losses to double after the system fails’’ (T_2_). Since buried HDPE geomembranes are estimated to have service life of many hundreds of years in ideal circumstances [14] it is likely that eventual leachate losses on average grow in an extremely slow pace during the post-closure period of time. It was therefore supposed that centuries would pass on average for leakages to intensify from the smallest- to the highest possible ones which were already measured on double lined systems. Spread of possible values for ‛T_2_’ was chosen in a way that scenarios with decreasing leakages across the post-closure time were considered to be realistic, too, when performing simulations (average value and standard deviation were both estimated to be 30 years).

Relationships between the inputs and long-term leakages were mathematically expressed as follows:

$$\begin{array}{c} \begin{array}{c} Q_{t}\;=\;\;0,\;\text{if}\;t\;<\;t_{\text{failure}}\;;\\ Q_{t}\;=\;\;Q_{0}\;\;\cdot \;\;\text{exp}\;\left[K\left(t\;\;-\;\;t_{\text{failure}}\right)\right],\;\text{if}\;\;Q_{0}\;\cdot \;\text{exp}\;\left[K\left(t\;\;-\;\;t_{\text{failure}}\right)\right]\;\;£Q_{\text{max}}\left[m^{3}/\text{year}\right]\text{and}\;\;t\geq t_{\text{failure}}\;;Q_{0}\;=\;q_{0}\;\cdot \;A\;;\\ Q_{\text{max}}=q_{\text{max}}\;\cdot \;A\;;\;K\;=\;\text{ln}2/T_{2} \end{array}\\ Q_{t}\;=\;\;Q_{\text{max}}\left[m^{3}/\text{year}\right],\;\text{if}\;Q_{0}\cdot \text{exp}\;\left[K\left(t\;-\;t_{\text{failure}}\right)\right]\;\;>\;\;Q_{\text{max}}\left[m^{3}/\text{year}\right] \end{array}$$

t_failure_ [years]: post-closure time which has to pass for composite liner system to fail
Q_0_ [m^3^/year]: initial leachate losses into the underground soon after liner system fails
q_0_ [lphd]: initial specific leachate losses into the underground soon after liner system fails [liters per hectare per day]
A [m^2^]: landfill footprint area
K [year^−1^]: first order rate constant describing increase of leachate losses after the system fails
T_2_ [years]: time needed for leachate losses to double after the system fails
Q_max_ [m^3^/year]: maximal possible leachate losses into the subsoil
q_max_ [lphd]: maximal possible leachate losses into the subsoil per unit area of landfill footprint

Wet-type landfills were assumed to leak twice as much as dry-types on average in order to consider greater possibilty for leachate collection systems to clog (potentially inducing eccessive water heads) and for high leachate temperatures to develop within the landfill interior (potentially inducing geomembrane failures and/or CCL cracking [25]).

Probability density distributions of values attributed to above mentioned inputs were presented in the companion articles [1,2]. It has to be taken in mind that input variables were quantified on basis of processed secondary data derived by performing monitoring and testing on landfills and experimental CLS systems which were considered to be ‘’modern’’ in the recent past. In general, these values do not represent well the characteristics of contemporary modern landfills. In other words, state-of-the-art landfills constructed in highly developed countries today undoubtedly outperform landfills which were lined with composite liner systems during the 1980s and 90s, due to

- advances of knowledge related to factors that influence long-term performance of composite landfill liners [15]
- advances in installation quality and construction quality assurance (CQA) practices (leakage rates greater than 50 lphd have decreased significantly in the past 20 years [20]); best available technology for locating leaks in geomembranes before they become a problem is geoelectric leak location methods, also known as liner integrity surveys; ideally, a bare geomembrane method would be used after geomembrane installation, then the dipole method would be used after the placement of cover materials
- greater durability and chemical resistance of geomembranes; HDPE geomembranes produced nowadays are extremely durable products, designed with service lives up to several hundreds of years under ideal conditions [14], [28], [29]
- greater percentage of double-lined landfills built today than in the past
- improvements in waste acceptance procedures and criteria for wastes to be disposed in landfills (e.g., [29]), which means, pollution potential per ton of received waste is much lower now than it was years and decades ago

On the other hand, in-place densities of waste are much bigger nowadays on average than they were decades ago. Consequently, waste stabilization rates are generally lower in contemporary modern landfills than they were in the older ones. Also, it has to be taken in mind that quality of landfill capping systems improved over the decades, not just the quality of bottom-liner-systems. Although short-term environmental risks diminished tremendously because of the above mentioned advancements, the same cannot be claimed when evaluating long-term environmental performance of state-of-the art landfills. One paradox exists which is usually ignored and can be in short presented as follows: (1) The better the long-term sealing efficiency of implemented cover systems → (2) the slower the rate of decline of the related pollution potential accumulated at these landfill sites → (3) the greater the long-term environmental risks. Consequently, not only average ‘t_failure_’ values would be estimated in terms of hundreds- rather than tens of years when referring to state-of-the-art conventional landfills, the same is true for ‘T_0.5_’ values as well. The problem is that these two inputs adversely affect outputs results. Therefore, when performing long-term risk assessment simulations, eventual pollution “events’’ would be shifted few hundreds of years further into the future, but scenarios with excessive release of pollutants into the environment would still be calculated by the model.

As opposed to state-of-the-art conventional (i.e., dry-type-) landfills, state-of-the-art bioreactor landfills demonstrate high rates of pollutant concentration decline in primary leachate after landfill closure (which means, relatively low average values are characteristic for the input ‘T_0.5_’), however, greater probability for CLS failure has to be considered, too, based on previous studies [15,25] (i.e., relatively low average values have to be attributed to the input ‘t_failure_’ as well).

#### Calculation of more complex outputs

Once probability distributions for the outputs ‘C_t_’ and ‘Q_t_’ are known, fugitive emissions of pollutants into the subsoil and their probabilities ‘QRP_t_’ can be acquired by calculating the product of the two:

$$\text{QR}P_{t}\;\;=\;\;C_{t}\;\;\cdot \;\;Q_{t}$$

QRP_t_ [kg/year]: Quantity of a reference pollutant released into the subsoil during the post-closure year ‘t’

All other outputs can be acquired by further processing already obtained simulated data for the parameter 'QRP_t_’. If the aquifer existed directly underneath the landfill, separated just by a narrow, permeable vadose zone, yearly fugitive emissions into the subsoil would be equal to annual discharges into the aquifer. Environmental permits would not be given to operators of such sites, however, the considered concept is adequate for modeling purposes, especially for quantitatively comparing long-term groundwater-protection efficiency of different types of sanitary landfills, which was the objective of the related articles [1,2]. For solving such-a-task the common environmental setting has to be presumed, anyway: it has to be as simple and risky as possible in order the expected differences would be clearly revealed. More thresholds and related levels of aquifer contamination were determined (defined as moderate-, severe- and irreversible-) rather than just one in order to analyze differences in environmental performance between landfill types from various aspects. In flowchart Fig. 1 both modeling pathways are shown, i.e. considering possibilities of either direct or indirect release of landfill-derived pollutants into the aquifer.

In realistic hydro-geo-environmental settings pollutant emissions into the subsoil are not equal to the related discharges into the aquifer. Correlations between the two can be potentially acquired after performing hydro-geo-environmental modeling (i.e., evaluating transport of pollutants and their fate in the environment before they eventually reach the aquifer and discharge into it as depicted below:

**Table:** 

Pollutant source		Pollutant pathway (hydrogeoenvironmental modeling)		Pollutant receptor
QRP_t (emissions into the subsoil)_	→	pollution attenuation within the hydrogeoenvironment	→	QRP_t (discharges into the aquifer)_

Environmental conditions at given compliance points would be eventually determined in this way. However, if the problem in consideration does not seem to be very important and/or complicated, the correlation between the two can be simply expressed by introducing an attenuation factor

$$\begin{array}{c} \text{QR}P_{t\;\;\left(\text{emissions}\;\;\text{into}\;\;\text{the}\;\;\text{subsoil}\right)\;}\cdot A_{F}\approx \text{QR}P_{t\;\;\left(\text{discharges}\;\;\text{into}\;\;\text{the}\;\;\text{aquifer}\right)}\\ A_{F}\ldots \;..\text{attenuation}\;\;\text{factor}\;\;\left(0\leq A_{F}\leq 1\right) \end{array}$$

If the aquifer lies directly below the landfill, separated just by a thin vadose zone, the two quantities appear to be equal (i.e., A_F_ ≈ 1). The equation also implies that in the case landfill hydrogeological setting was ideal (i.e., A_F_ ≈ 0), the aquifer would not be affected even if landfill's environmental-protection performance was extremely bad.

Long-term environmental risk can be defined as the likelihood that an aquifer will be contaminated because of leakage from a landfill. Groundwater thresholds are normally expressed in pollutant concentration units. However, if the aquifer threatened by the landfill is already well researched, a hydrogeologist is in principle able to define threshold values directly in terms of required reference pollutant discharges into the aquifer which would invoke such already prescribed pollutant concentration levels within the groundwater, e.g.

$$\begin{array}{c} \text{QR}P_{t\;\;\left(\text{threshold}\;\;\#1\right)}\;\;\leq \;\;\text{MLP}\;\;\leq \;\;\text{QR}P_{t\;\;\left(\text{threshold}\;\;\#2\right)}\leq \;\;\text{SLP}\;\;\left[\text{kg}\;\;\text{of}\;\;\text{reference}\;\;\text{pollutant}\;\;/\;\;\text{year}\right]\\ \text{CUMQR}P_{t\;\;\left(\text{threshold}\#3\right)}\;\;\leq \;\;\text{ILP}\;\;\left[\text{kg}\;\;\text{of}\;\;\text{reference}\;\;\text{pollutant}\right] \end{array}$$

The above mathematical expressions can be articulated as delineated below:

• If/when the calculated quantity of reference pollutant discharged into the aquifer during the post-closure year ‘t’ is greater than the lower threshold value but smaller than the higher threshold value, the acquired level of groundwater pollution is considered to be moderate during that particulate year (moderate level of pollution, or MLP).
• If/when the calculated quantity of reference pollutant discharged into the aquifer during the post-closure year ‘t’ exceeds the higher threshold value, the acquired level of pollution is considered to be severe during that particular year (severe level of pollution, or SLP).
• If/when the overall amount of reference pollutant cumulatively discharged into the aquifer during the post-closure period of time until the year ‘t’ exceeded the predisposed threshold, the aquifer itself can be considered to be polluted, not just the related groundwater (i.e., an ‘irreversible’ level of aquifer pollution was reached, or ILP).

Environmental risks can be quantitatively assessed by calculating the probabilities that a given aquifer will be moderately, severely or irreversibly polluted due to landfill- derived impacts (i.e., P_MLP_, P_SLP_ and P_ILP_ have to be calculated). According to the definitions described above, moderate and severe levels of pollution are considered to be temporary, reversible conditions as opposed to irreversible level of pollution, which is considered to be indefinitely long lasting condition. Moderate level of pollution is reached during the post-closure year when annual pollutant discharges surpass the given lower threshold and lasts until the year when the pollutant discharges fall below that threshold once again.

Probability for an aquifer to become polluted is related to the considered length of time after landfill closure. The longer the time, the greater the probability. Therefore, time- lengths required for moderate-, severe- and irreversible levels of pollution to be reached and for moderate- and severe levels to end are calculated together with their probabilities. In the related research- and data-description articles [1,2] these outputs were labeled as MLP_starting_, SLP_starting_, ILP_starting_, MLP_ending_ and SLP_ending_, respectively. Overall probability (i.e., P_MLP_, P_SLP_ or P_ILP_) can be calculated only when considering the entire duration of time until a residual threat appears to be present at the site, i.e., until the particular landfill exhibits sufficient pollution potential to harm the aquifer.

**Fig. 2:**
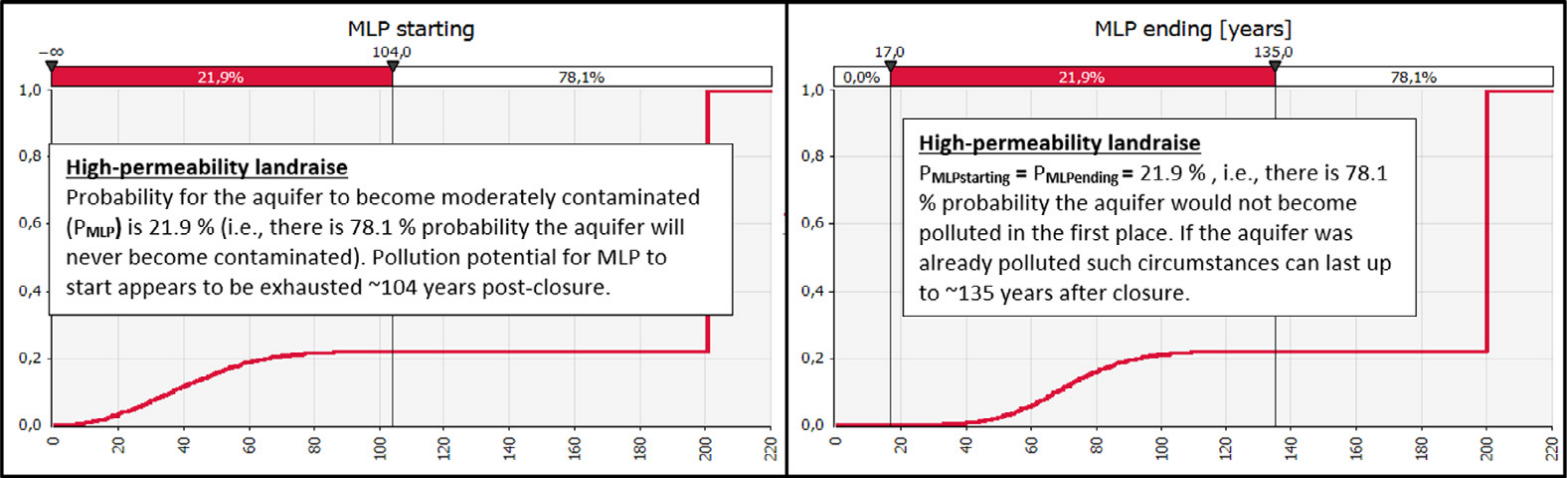
Comparative demonstration of the related MLP_starting_ and MLP_ending_ graphs (extracted from the companion data- description article [2]).

The output MLP_starting_ gives probability distribution of values for a parameter “post-closure time needed for MLP to begin’’ (where time is given in years and probability in percents). Cumulative probability for MLP to start rises over the passage of time until reaching a plateau. It can not rise any more once the landfill is stabilized/ detoxified and poses no threat to the environment. MLP_starting_ output results are best represented graphically by a cumulative probability curve as shown in Fig. 2. Therefore, by acquiring probability distribution of outcomes for the output MLP_starting_, the overall (total) probability for MLP is also derived, where ’P_MLP_’ is given as a discrete value expressed in%.

The output MLP_ending_, on the other hand, gives probability distribution of outcomes for the parameter “post-closure time needed for MLP to end’’ as a result. ‘MLP_ending_’ cumulative probability curve reaches the plateau at a later post-closure year than the related ‘MLP_starting_’ curve does, however, the calculated overall probability is the same for both of the curves as it should be (P_MLPstarting_ = P_MLPending_) – see Fig. 2. By analyzing ‘MLP_ending_’ statistical data one can define the post-closure year when landfill does not pose reasonable threat to the aquifer any more with some high degree of probability, e.g. 90%. Duration of average time required for these conditions to be met can differ by several hundreds of years when comparing post-closure environmental performance of different types of sanitary landfills. Pollution potential of a landfill to contaminate the adjacent aquifer further in the future is therefore depleted at this point in time. In other words, calculated reference pollutant annual discharges fall below the levels which can potentially pose a threat to local groundwater resources, i.e.,

$$\begin{array}{c} t_{\text{QRPt}}\;\;\geq \;\;\text{ML}P_{\text{ending}}\left(P\;\;=\;\;90\%\right)\\ \text{ML}P_{\text{ending}}\;\;=\;\;t_{\text{QRP}\;\;\text{threshold}\;\;\#\;1\;\;\text{reached}\;\;\text{during}\;\;\text{the}\;\;\text{post}-\text{closure}\;\;\text{period}\;\;\text{of}\;\;\text{time}\;\;\text{characterized}\;\;\text{by}\;\;\text{declining}\;\;\text{pollution}\;\;\text{discharges}}\\ \;\;\;\;\left[\text{years}\right] \end{array}$$

However, landfill can be considered to be fully stabilized/detoxified considering other criterions, too, e.g.

$$\begin{array}{c} t_{\text{QRPt}}\;\;\geq \;\;\text{SL}P_{\text{ending}}\left(P\;\;=\;\;99.5\%\right)\\ \text{SL}P_{\text{ending}}\;\;=\;\;t_{\text{QRP}\;\;\text{threshold}\;\;\#2\;\;\text{reached}\;\;\text{during}\;\;\text{the}\;\;\text{post}-\text{closure}\;\;\text{period}\;\;\text{of}\;\;\text{time}\;\;\text{characterized}\;\;\text{by}\;\;\text{declining}\;\;\text{pollution}\;\;\text{discharges}}\\ \;\;\;\;\left[\text{years}\right] \end{array}$$

Overall groundwater pollution potential which exists immediately after the landfill is closed can be retrospectively expressed in terms of cumulative quantity of a specific pollutant which can be potentially released into the aquifer considering infinite post-closure time:

$$\text{CUMQR}P_{\text{max}}=\sum \text{QR}P_{t\;}\left[\text{kg}\right]\;;\;\;0\leq t\leq \infty$$

As already explained above, this quantity can be acquired accurately enough when considering post-closure time which is at least equal to the time needed for calculated emissions to fall below the lower threshold:

$$\text{CUMQR}P_{\text{max}}\approx \sum \text{QR}P_{t}\left[\text{kg}\right]\;;\;\;0\leq t\;\;\leq \;\;\text{ML}P_{\text{ending}}\left(P\;\;=\;\;90\%\right)$$

Spreadsheets which were designed to derive all of the above described outputs in order to compare environmental performance of different types of landfills based on premises outlined in the companion research article [1] are presented in Section *``Construction of spreadsheet models”*. The related outcomes are graphically presented and compiled in the companion data- description article [2].

## Derivation of probability distributions for the inputs ‘C_0_’ and ‘T_0.5_’

Biodegradability of organic content of MSW and heavy compaction of waste after its placement makes the landfill an anaerobic environment, giving many similarities to generated leachates compositions among the sanitary anaerobic landfills in general; a strong relationship exists between the state of refuse decomposition and its associated leachate characteristics [30,31]. Semiaerobic landfill environment on the other hand generates leachates with their own distinct characteristics [32].

Likewise, strong relationship exists between the rate of decline in primary-leachate pollutants- concentrations during the post closure time and landfill type/subtype involved in generating this trend as written below:

1. T_0.5 dry-type landfill_ >> T_0.5 wet-type landfill_

The related factor: refuse moisture content [33]

2. T_0.5 non-flushing landfill_ >> T_0.5 flushing landfill_

The related factor: rate of the pollutants washout [34]

3. T_0.5 non-treated leachate recirculating landfill_ >> T_0.5 ex-situ treated (e.g., nitrifyed) leachate recirculating landfill_

The related factor: recirculated liquids composition [35]

4. T_0.5 anaerobic landfill_ >> T_0.5 (semi)-aerobic) landfill_

The related factor: environmental conditions occuring within the landfill body [32]

In other words, ranges of probable values for typical pollutants are characteristic for landfills of particular types and ages. By acquiring general information regarding the history of a particular landfill one readily gets a clue about its primary leachate composition.

A particular landfill can be at the same time e.g. anaerobic, wet-type, non-flushing, etc. It can be a modern, highly engineered facility or a simple above ground waste deposit. Characteristic values for parameters ‘C_0’_and ‘T_0.5_’ can be estimated based on this and other information. The problem of uncertain inputs is usually considered by assigning them with probability distributions of values. In general, the better the particular landfill or a landfill group is defined, the more precise these estimates can be. Values tend to cluster together around the averages which are typical for particular landfill types. Probability distributions for the inputs are more spread out when environmental performance of landfill types are evaluated (rather than environmental performance of particular landfills), because heterogeneity factor has to be considered, too, not just the uncertainty factor.

Therefore, selection of probability distributions is meant to be based on comprehensive knowledge related to characteristics of different types of landfills. Some experts will not agree with probability distributions estimates given by other experts or professionals, but not in any dramatic way, since basic facts cannot be changed.

### Representativeness of ammonia nitrogen as a reference pollutant

The focus of the companion research study [1] was put on comparing long-term environmental performance of different types of sanitary landfills. For practical reasons, only the most representative aqueous pollutant was considered to perform comparisons, i.e. the one which is characteristic for sanitary landfills after their closure. Among other dissimilarities, leachate pollutants differ according to their characteristic timelines of occurence in relation to the succession of characteristic phases of waste decomposition. If (for example) volatile fatty acids (VFA)'s were used as a representative parameter in order to compare long-term environmetal performance of different landfill types, no differences would be detected, since these pollutants are generated during the acidogenetic phase, i.e., by degradation of freshly disposed MSW. These pollutants are almost not even present within the leachate after landfill closure. Similarly, the content of heavy metals in landfill leachates is in general already low during the stable methanogenic phase as a result of alkaline conditions occuring within the buried waste and attenuating processes (sorpion and precipitation) that take place within the disposed waste [30]. Comparative landfill-simulating tests showed either no major differences in leachate heavy metals concentrations between anaerobic, semi-aerobic and aerobic bioreactors [36] or better performance of semi-aerobic reactors because they more readily act as a final sink for heavy metals due to fast stabilization of organic matter into humic substances [37]. At high-permeability-landraise (HPL) research site [1], the concentration of heavy metals in primary leachate was always very low, too.

Leachate pollution index [38] or leachate toxicity could be better parameters for characterizing leachate pollution potential, however, for purposes of comparing environmental performance of different types of landfills in general these parameters seem to be too complex in order to convincingly attribute the related input variables ‘C_0’_and ‘T_0.5_’ with probability distributions of values.

Ammonia nitrogen is a typical persistant pollutant, one of the most representative ones for purposes of analysing long term environmental performance of different types of landfills. Many renowned reserchers acknowledged that (e.g., [30,31]). This pollutant does not seem to follow much faster decreasing trends characteristic for many other leachate pollutants, such as biological oxygen demand (BOD).

### Derivation of ‘C_0’_and ‘T_0.5_’ as modeling inputs in the related research study

When referring to the research article [1], probability distributions of ammonia-nitrogen values attributed to input variables ‘C_0’_and ‘T_0.5_’ were obtained by qualitatively processing data related to real-world landfills but also by considering information derived from laboratory and pilot scale experiments. Different liquid-to-solid (L/S) ratios and different scales of heterogeneity which exist between laboratory-, pilot-scale- and realistic landfill- environments [39], [40] were taken into account.

Explanation:

Literature specifically targeting ‘C_0’_and ‘T_0.5_’ values does not exist. On the other hand, there is a vast body of literature which provides theoretical and practical information about the subject, especially with respect to NH_4_ – N characteristics as a reference pollutant (e.g., explaining why ammonia nitrogen is so persistent pollutant in anaerobic landfills but not in semiaerobic landfills and certain types of leachate-recirculating and/or flushing landfills). It is true, concepts of different types of sanitary landfills are vaguely defined, but the nature of the work is intrinsically based just on such kind of imprecise information. When gathering information regarding leachate composition from real life landfills one should be aware that these systems are almost never clearly defined, at least not from all of the relevant aspects. However, this does not mean such information is irrelevant. It is up to the researcher to select and interpret these data appropriately and to connect this information with findings obtained from laboratories and pilot-scale studies where systems were precisely defined (Table 1). Uncertainty is considered during the step when attributing inputs with probability density functions. Explaining how input data were acquired is an essential part of the applied approach. Similar approaches are used in other fields, too, when dealing with situations which are subject to uncertainty (e.g., [11,13]).

**Table 1:** Results from the literature performing nitrogen removal tests.

LSR (landfill-simulating-reactors) and pilot scale tests (values in parentheses: NH_4_^+^- N concentration in leachate [mg/L])	
Plexi glass reactors with 20 kg waste taken from Modena landfill. Leachate after ten weeks: anaerobic reactor (water addition, no recirculation): ~(800 → 200); aerobic reactor (water addition, no recirculation): ~(800 → 10) aerobic reactor (leachate recirculation): ~(1100 → 10)	[40]
Waste samples from Kuhstedt landfill before and after 6 years of low-pressure aeration. Leachate obtained from waste samples (L/S ratio = 0.12): Waste before aeration: 345; waste after aeration: 54	[32]
Experiment in 7 m^3^ tanks, clean water added, no leachate recirculation. Anaerobic tank: 1300 → 700 in 3 y. Aerobic tank: 1300 → ~ 0 in 1 year	[41]
Experiment in Φ1.2 m, 7.5 m high lysimeters; clean watter addition; 16 months period. Anaerobic: ~1500 → 700; Semiaerobic: ~1200 → 200	[42]
Experiment in two phases (dry and wet) simulating tropical climate conditions; 8 lysimeters (φ 0.24 m, H 1.0 m); 4 semiaerobic, 4 anaerobic (half of them with high% putrescible fraction waste); no recirculation. Anaerobic: (10th day → 95th day): ~ 1200 → 1100 Semiaerobic: (10th day → 95th day): ~800 → 0 Cumulative outflow of NH_4_ – N with the leachate (190 days): Anaerobic: ~ 25 g; semiaerobic: ~3 g.	[43]
Lysimeters (Φ 0.9 m, H 2.7 m), simulating tropical climate; Semiaerobic 1 (density 640 kg/m^3^), semiaerobic 2 (density 770 kg/m^3^), anaerobic 1 (density 730 kg/m^3^, 50% flooded), anaerobic 2 (density 720 kg/m^3^, 100% flooded). No recirculation. Day 120 → day 650. TKN was measured. Anaerobic 1: ~ (1250 → 750), anaerobic 2: ~ (2450 → 1100) . Semiaerobic 1: ~ (1250 → 50), semiaerobic 2: ~ (2750 → 100) .	[44]

Leachate quality data is widely available from all around the world (sometimes describing particular landfills vaguely as young, mature or old, small or large, dry or wet, controlled or uncontrolled, etc.). Information about the spread of possible values for parameters ‘C_0’_and ‘T_0.5_’ has largely derived from such kind of sources as presented in Table 2.

**Table 2:** Ammonia nitrogen concentration ranges characteristic for some landfills around the world.

NH_4_^+^- N concentration of the primary leachate [mg/L]) Waste is usually heavily compacted in modern landfills and the milieu in their interior appears to be very anaerobic. Higher values for NH_4_^+^- N (higher than ~1200 mg/L) gathered from waste disposal sites all around the world largely apply to anaerobic landfills during the time they were still young and/or if ammonium was not eluted out of their bodies in large enough quantities yet.	
Acidofilic phase landfills: 2–1030; Final maturation phase landfills: 6–430	[45]
Old landfill of Legnago: 900–3500 Calancoi closed landfill: 1500–1800	[46]
Bioreactor anaerobic landfills: 100 –500, average 740	[47]
Landfills in Germany: 30–3000; mean 750	[48]
104 small, old unlined Danish landfills, on average closed for some 17–18 y: Generally below-ground piles (~anaerobic): 17.5–83.9 Generally above-ground piles (~semiaerobic): 1.3–5.9	[49]
Upper bound values: 5 y old landfill: 800; 10 y old: 700; 20 y old: 590; 30y old: 580; 40y old: 570	[50]
32 closed, lined Austrian/Swiss landfills; (on average, 16 y post-closure time has already expired): 1.1–6200; mean 1045	[6]
Ajdovščina high-permeability landraise (passive semiaerobic above ground landfill): 450 (immediatelly after closure of the 1. sector) → 75 (8 years post closure); decline continues to this day	[9]
Landfills: Montreal 179; Montevideo 1470; Thessaloniki 3100; Hong Kong 1190–2700; Kyungjoo (Korea) 1682; Shenzen (2 y old) 2090	[51]
Shangai Laogang landfill, fresh leachate (operating landfill section): 4632; semi-mature leachate (5 years old landfill section): 2197; mature leachate (11 years old landfill section): 1388	[31]

“Average/mean)” or “most probable” values (i.e. values with greatest probabilities of occurrence within the selected probability distributions) were mostly estimated after processing large amounts of secondary data which are only indirectly related to parameters ‘C_0’_and ‘T_0.5_’. The main sources were: Laner [6] for composite-bottom-lined landfills (aka “modern’’ landfills), Kjeldsen and Christophersen [49] for dumpsites and Madon [1,9] for HPL's as described in Table 3.

**Table 3:** Derivation of ‘C_0’_and ‘T_0.5_’ average inputs estimates.

‘C_0_’: primary leachate ammonia-nitrogen concentration immediately after landfill/ landfill- compartment closure. ‘T_0.5_’_:_ Half-life period (refers to post-closure time required for leachate ammonia-nitrogen concentration to reduce to half of its initial value)	

Input parameter	The most expected (mean) value

‘C_0’_for modern landfills (dry and wet types)	µ = 1200 mg/L

Explanation: Data from Laner [6] refer to 32 closed Austrian and Swiss landfills which were bottom lined with composite liner systems (= modern landfills according to definition applied in this work). On average, these 32 landfills were already closed for 16 years in 2008. NH_4_^+^- N ranged from 1.1 to 6200 mg/L in 2008 (mean value = 1045 mg/L). Based on this information it seems to be reasonable to choose 1200 mg/L as a proper “average” value for the input parameter ‘C_0_’. Landfills (or landfill cells) normally do not operate as bioractors before their closure (in order to avoid excessive fugitive emissions of methane from the uncovered active areas, etc.). The same average value was therefore selected for all modern landfills, i.e. dry- and wet- ones. *The most common values for ammonia nitrogen concentrations in anaerobic landfills during the stable methanogenic phase (landfills are usually closed when they go through this phase) found in the literature appear to be within the range 450 mg/L – 800 mg/L rather than >1000 mg/L. However, ammonia nitrogen leachate concentrations tend to decline fast after landfill closure when the values are higher than 1000 mg/L (i.e., “half-lives” tend to be shorter than average) than later when the values are within the more common range between 450 and 800 mg/L (“half-lives” tend to be longer than average). Therefore, trying to find the right selection of average values for ‘C_0’_and ‘T_0.5_’ as a set is more important than trying to find the correct averages for ‘C_0’_and ‘T_0.5_’ separately.	

‘T_0.5_’ for modern landfills, dry type.	µ = 40 years.

Explanation: Landfill- stabilization- progress after landfill closure manifests itself in different ways (decline in annual quantity of generated landfill gas, decline in respiration rates measured on solid waste samples taken from the landfill, decline in concentration of aqueous pollutants in primary-leachate samples taken at the bottom of the landfill, decline in settlements rates, etc.). All these phenomena are interrelated, but half-life periods are not equal and the related stabilization rates can be only vaguely approximated as being of the pseudo-first order rate. Historically, the concept was mostly used to model methane generation rates. E.g., US EPA in its document AP-42, fifth edition [52] set forth default values for first-order decay rate constant to be used in its LandGEM model for conventional landfills: *k* = 0.04 (T_0.__5_ ≈ 17 years) for wet climates and *k* = 0.02 (T_0.__5_ ≈ 35 years) for dry climates. Modern dry-type landfills are sealed at the top when the particular compartment is filled with waste, therefore, T_0.__5_ ≈ 35 years would be a good first estimate for characterizing rate of decline in the intensity of stabilization processes taking place in ‘’dry-entombment’’ landills. However, no major biological pathways for ammonia nitrogen removal exist within the anaerobic landfill ([53,35]), consequently, the related stabilization process is inherently very limited. The pollutant can be removed almost exclusively by the washout process, but leachate generation rate decreases rapidly after a dry-type landfill is capped. Therefore, half-life period tends to be much longer than 35 years when ammonia nitrogen is considered as a reference pollutant. However, in reality many composite-liner caps do leak a little bit immediately after the landfill was closed [54] and leakings rise slowly in the long term. The paradox is that leaky covers eventuate faster decline in concentrations of pollutants within the primary leachate (shorter ‘T_0.5_’) resulting in better long-term groundwater protection performance of landfills with defective final covers. Based on the contemplations outlined above the arbitrarily selected average value of 40 years for ‘T_0.5_’ does not seem to be conservative at all. Paradoxically, if the value was set to be 60 years instead of 40 years in the comparative study [1] (i.e., if landfill covers with better sealing characteristics were supposed to be installed on average), dry-type landfills would have environmentally performed even worse when compared to other landfill types executing long-term risk assessment simulations.	

‘T_0.5_’ for modern landfills, wet type.	µ = 7 years.

Explanation: Gas decay constant value of *k* = 0.1 (T_0.__5_ ≈ 7 years) was proposed to EPA as a default value for predicting long-term gas generation rates in bioreactor landfills [55]. Research performed by Tolaymat et al. [56] confirmed such proposal to be reliable. However, similar rates of decline are not necessarily characteristic when predicting steady fall in concentrations of persistent aqueous pollutants within the primary leachate. For example, ammonium nitrogen concentration cannot be abated down just by performing recirculation within an ordinary anaerobic environment [53,35]. However, ammonia- as well as total- nitrogen can be readily removed by • recirculating *ex-situ* treated (nitrified) leachate before being inserted back into the landfill interior [35] • recirculating leachate within an aerated or hybrid (anaerobic/aerobic) bioreactor- landfill system [53] Rapid rates of pollutant concentration decline can be sometimes observed in non-bioreactor landfills, too. Persistent pollutants can be namely abated down just by washing them out of landfill. Conventional modern landfills which are situated in humid climate and are covered only with local earthen materials can be also considered to be wet-type landfills. According to statistical analysis by processing raw leachate parameters- data related to 32 Austrian and Swiss landfills already closed for a long-time [6] it was revealed that chloride and ammonium concentrations within the primary leachate on average decreased quite rapidly and at a similar rate (T_0.__5_ ≈ 7 years). Chloride is perhaps the most typical persistent aqueous pollutant characteristic for MSW landfills. This parameter can be abated down only by means of washing it out of the landfill unlike ammonium nitrogen, which behaves as a persistent pollutant only in strictly anaerobic environment as mentioned above.It has to be pointed out that the studied group of landfills • were bottom-lined with composite liner systems [6] (therefore characterized as modern landfills according to the categorization described in Section ``*Types of sanitary landfills'**'***) • did not practice post-closure leachate recirculation [6] (therefore, they were not operated as bioreactors) • were capped mostly with local earthen materials (which could not have prevented part of the precipitation to enter the landfill; this was a prevalent way of covering landfills during the 1980s and 90s, anyway) • were of an anaerobic type (otherwise, ammonium and chloride would not have demonstrated so similar long-term declining rates) Modern bioreactor landfills are likely much more densely built than the studied group of old Austrian landfills on average (therefore, in general, more difficult to be stabilized). Based on information presented above it is reasonable to select T_0.__5_ ≈ 7 years as an average value for wet-type landfills as a whole.	

‘C_0’_for high-permeability landraises (HPL's)	µ = 450 mg/L

Explanation: Semiaerobic milieu provides conditions for nitrification/ denitrification to occur simultaneously within one landfill cell rather than requiring two separate cells containing two different in-situ environments, i.e. anoxic and aerobic [53]. High permeability landfill is aerated passively already during the operational phase, that's the reason why ammonium can not build up to reach high concentrations within the primary leachate. The value of 450 mg/L was selected based on Ajdovščina “prototype landfill” data.	

‘T_0.5_’ for high-permeability landraises.	µ = 3.5 years

Explanation: The value of 3.5 years was selected since it is characteristic for a “prototype HPL“ where the corresponding author performs research. Half-life period could have been shortened even more by intensifying landfill flushing operations. Since no relevant groundwater bodies exist in the vicinity (the most vulnerable part of the environment appear to be natural surface waters) such measures would not be justifiable.	

‘C_0’_and ‘T_0.5_’ for above-ground dump sites	µ_1_ = 250 mg/L; µ_2_ = 3 years

Explanation: An uncontained above- ground dump site (as defined in Section ``*Types of sanitary landfills”*) is basically an uncontrolled version of a HPL- type of landfill, on average even less compacted, sanitary covering more poorly applied, etc. (in other words, such type of “landfill” should be more aerobic than a HPL on average). Therefore, average ‘C_0’_and ‘T_0.5_’ values should be somewhat lower when compared to those which are characteristic for HPL's. The rounded average value of 250 mg/L has been derived by analyzing data collected from 106 old unlined Danish landfills [49] assorted in four groups in regard to where leachate monitoring wells were screened (labeled as group A, B, C and D, respectively). Group A was largely represented by below-ground disposal sites with wells screened in the saturated waste layers - 65 of them) and Group C was mostly represented by above-ground waste piles with wells screened in the underlying saturated geological layers - 103 of them). Due to this fact the possibility of significant leachate dilution was expected to exist for the group C, but not for the group A. Comparing data of chloride concentrations (a typical non-degradable pollutant) between groups A and C the ratio appeared to be 1.52, probably representing the dilution effect. However, when comparing ammonium concentrations between these two groups of dumpsites (ammonium is a persistent pollutant at anaerobic sites but a decaying pollutant at semiaerobic sites), the ratio was 14.1. Even if we attribute factor 2 to the dilution effect, the factor of 7 still remains to be attributed to ammonium biodegradation effect in semi-aerobic landfills (therefore, on average, 7-times smaller ammonia concentrations appear to be present within the leachate derived from passively aerated above-ground piles than from below-ground, anaerobic waste piles). Eliminating dilution effect, the average value for ammonium-nitrogen in group C would be some 7.2 mg/L. From the graph demonstrating sodium concentration as a function of landfill age in old Danish landfills [49] the average half-time period due to wash-out effect can be roughly obtained: T_0.__5_ *≈* 20 years. Therefore, ammonium concentration half-time characteristic for above ground dumps should be ~seven times shorter (i.e., T_0.__5_ *≈ 3* years on average). Considering that the evaluated Danish landfills were already closed for 17–18 years on average, ‘C_0’_value of ~250 mg/L can be acquired (first order rate equation calculated backwards). The corresponding author took leachate samples from two small abandoned above-ground dumps situated on an impermeable terrain in Vipava Valley (Slovenia) years ago and acquired similar C_0_ values.	

## Construction of spreadsheet models

As an add-in to Microsoft Excel, @RISK software provides all the necessary tools for setting up, executing, and viewing the results of risk analyses [11]. Excel-style menus and functions are used to construct a spreadsheet model. Distribution functions can be added to any number of cells and formulas throughout worksheets. These distribution functions are invoked only during a simulation. In normal Excel operations, they show a single cell value, just as in Excel without @RISK. Both Monte Carlo and Latin Hypercube sampling techniques are supported, and distributions of possible outcomes can be generated for any cell or range of cells in the spreadsheet model.

@RISK program graphs probability distributions of possible outcomes for each @RISK output cell. @RISK graphics include:

• Relative frequency distributions- and cumulative probability curves
• Summary graphs for multiple distributions across cell ranges (for example, a across a row of time series values)
• Statistical reports on output distributions
• Probabilities for target values in a distribution

All the necessary information related to software capabilities and usage is provided in the related User's Guide [11].

**Fig. 3:**
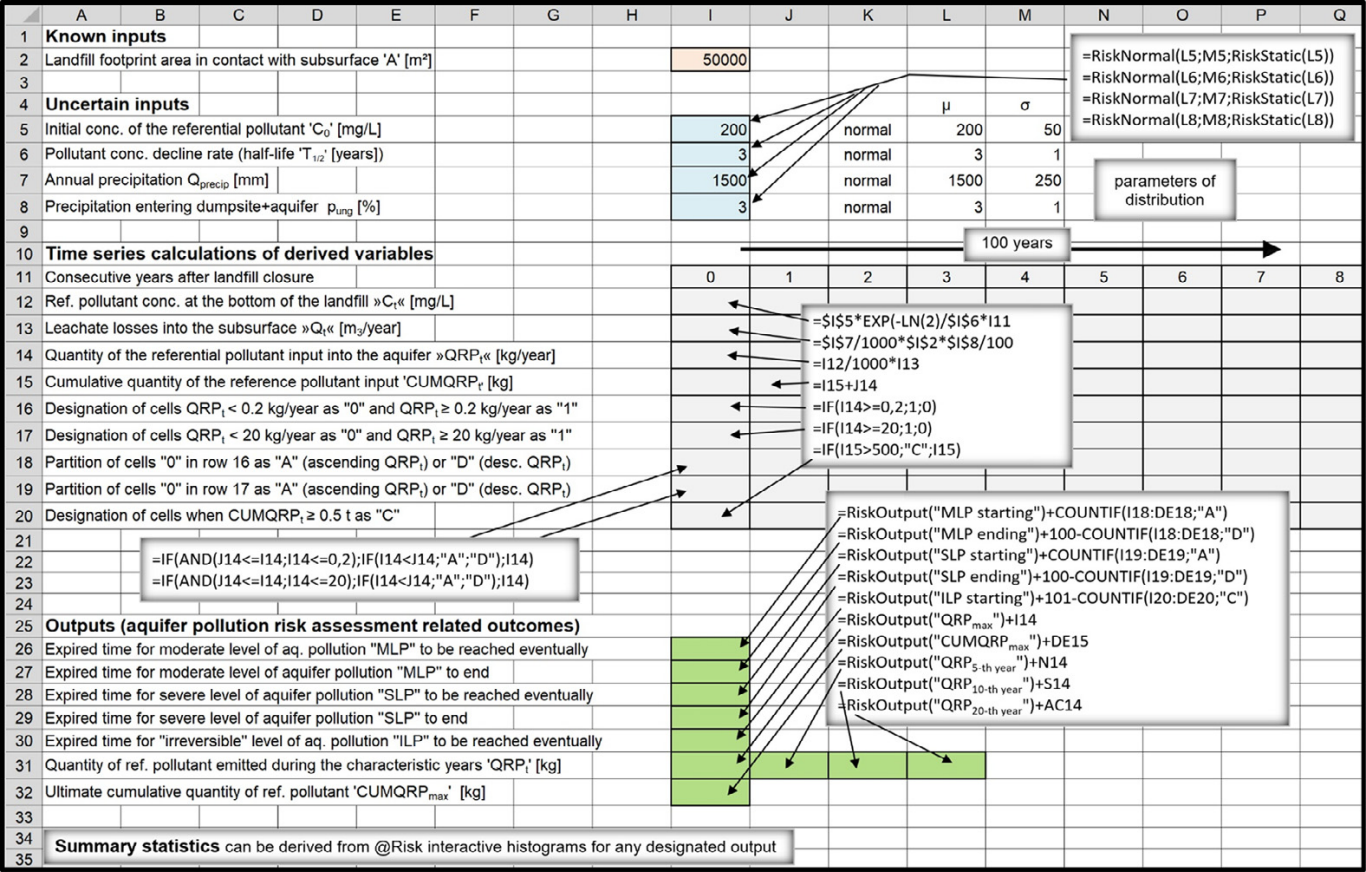
Above-ground dump site spreadsheet model.

In order to assess long-term environmental risks characteristic to four types of sanitary landfills based on premises described in the companion research article [1], four spreadsheet models were constructed each one representing one landfill type (Figs. 3, , , –6).

**Fig. 4:**
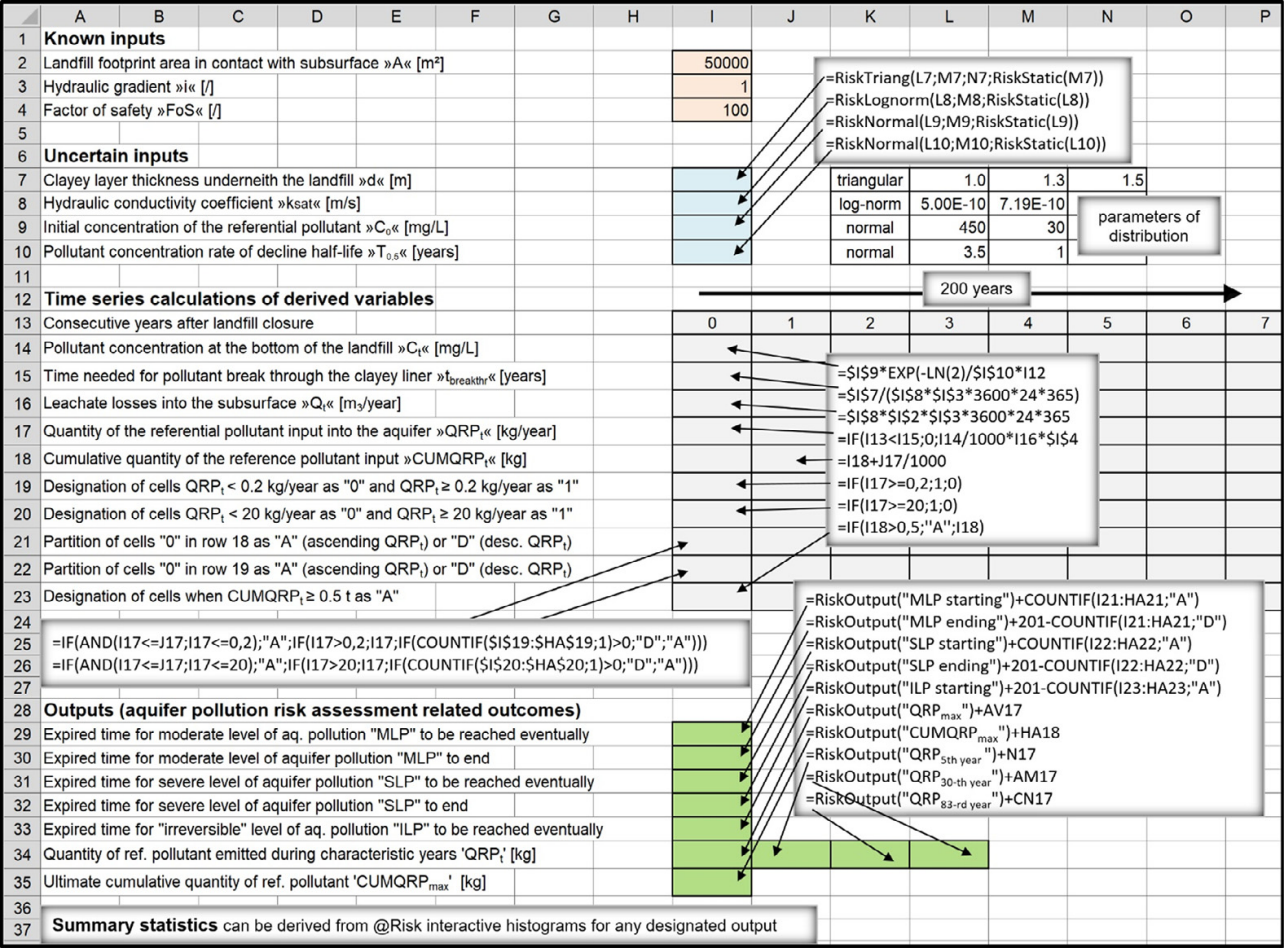
High-permeability landraise spreadsheet model.

**Fig. 5:**
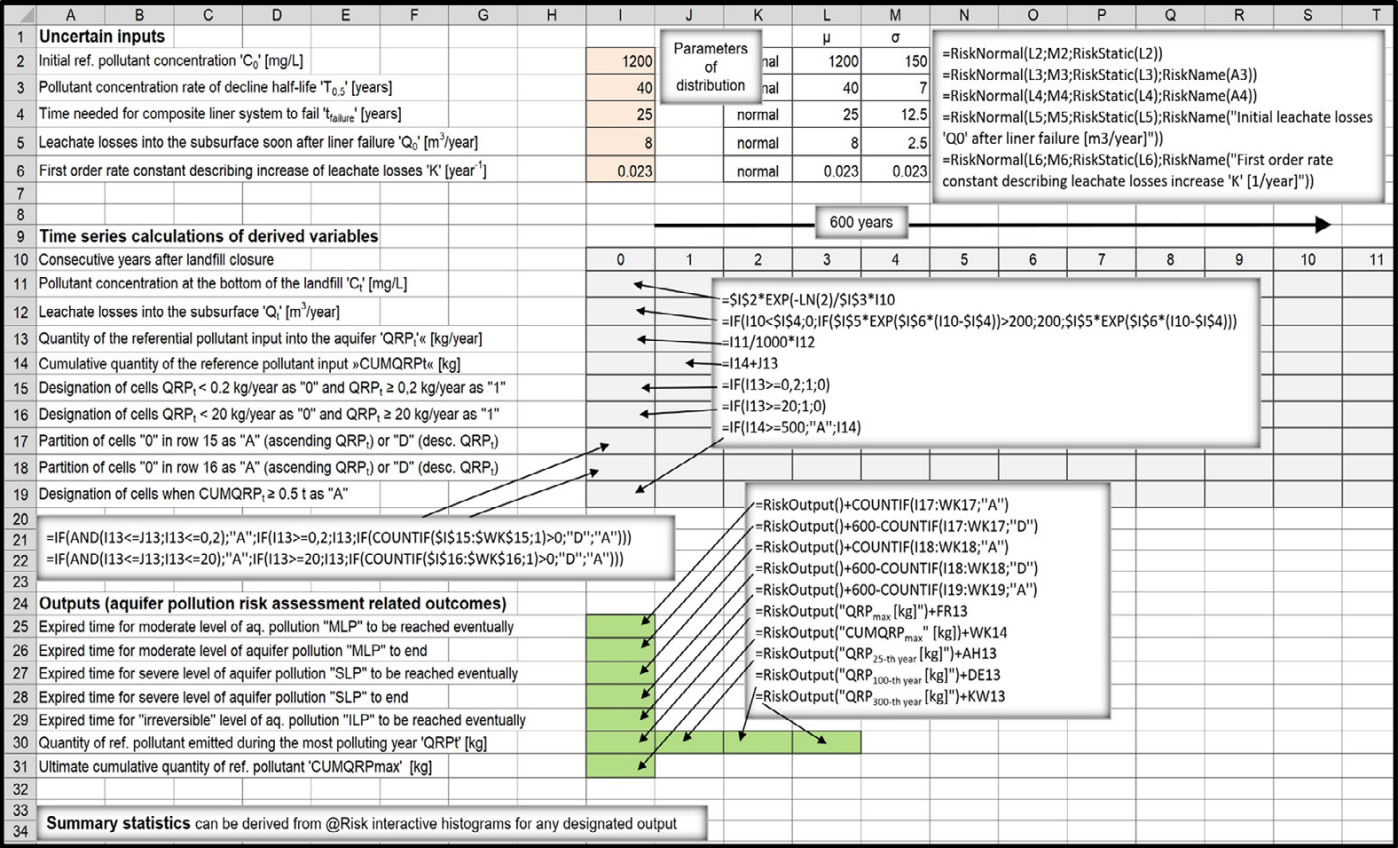
Modern dry-type landfill spreadsheet model.

**Fig. 6:**
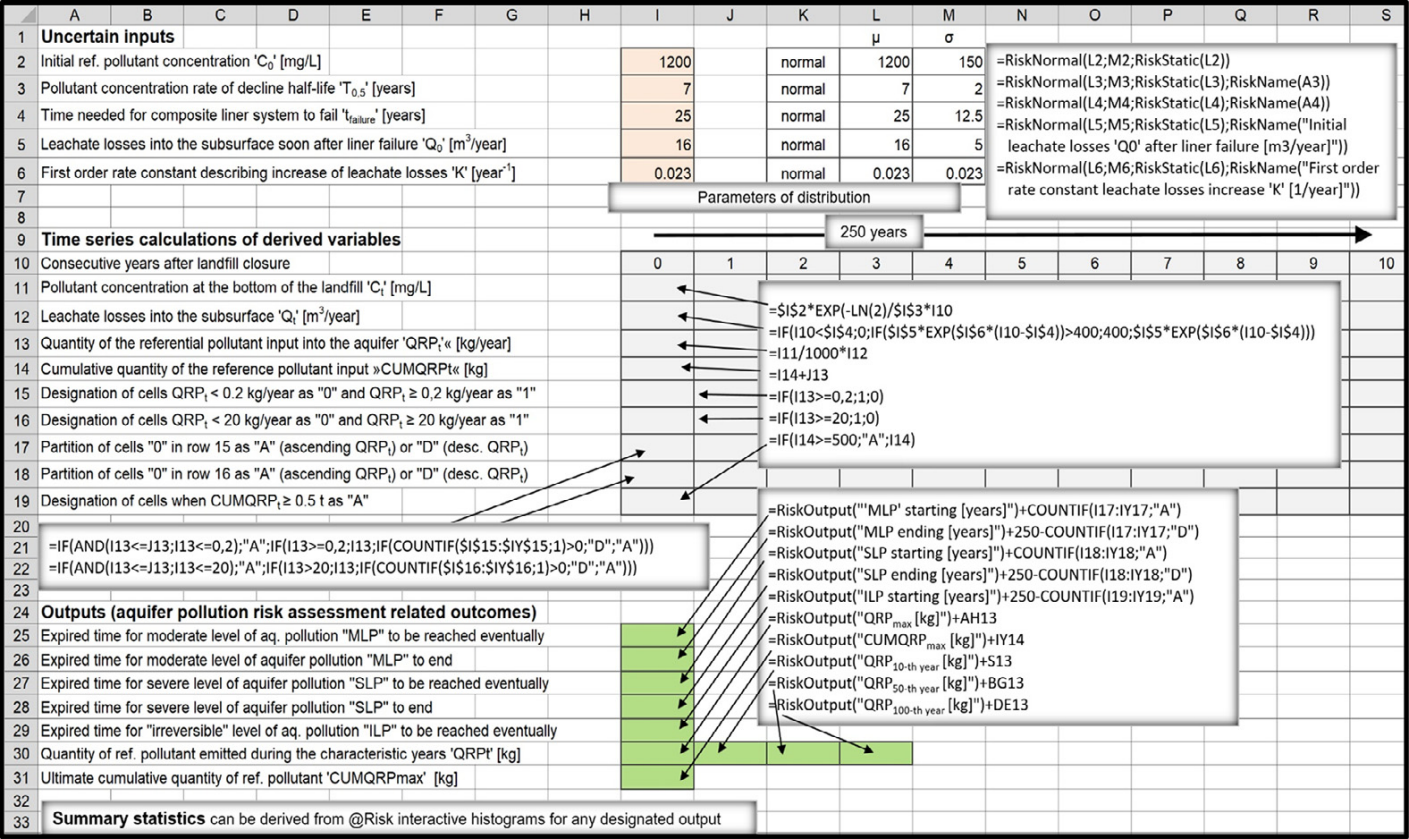
Modern wet-type landfill spreadsheet model.

The four landfill types were modeled as if being individual landfills occupying equal footprint areas in contact with the subsoil (5 hectares). During the initial phase (when attributing input variables with probability density functions) attempts were made to consider all the detected heterogeneities, complexities and uncertainties which are characteristic of different landfill types to be included into the model. Landfills were compared based on size-equivalence criterion (see Section ``*Functional-equivalence problem performing comparative risk assessments”*), therefore, all landfills representing antagonistic types were considered to have approximately equal capacities (~500.000 t). All were placed in the same hydrogeological and hydrological setting (humid climate, thin semi-permeable vadose zone was considered to separate landfill subgrade from the water table). Consequently, large part of annual precipitation was supposed to be transformed into surface run-off, but weak leakages typical for lined landfills were supposed to result into an unmittigated transport of pollutants all the way down to the aquifer. Outputs- results are presented in the related research- and data description articles [1,2].

The method is not intended to be fixed once and for all. More sofisticated formulas can be build for distinctive purposes, etc.

## Functional-equivalence problem performing comparative risk assessments

In general, overall risk assessment setting consists from three consistuent parts, separately defining the source of a potential hazard, pathways by which the damage may occur and the receptor of a potential hazard. When reffering to the case described in the research article [1], the

1. source was represented by a 5 ha, 500.000 t large closed sanitary landfill situated in a humid environment
2. pathway was represented by a semi-permeable vadose zone lying underneath the landfill, and
3. receptor was represented by a thoroughly researched aquifer lying underneith the landfill and the vadose zone

Great part of the eventual ambiguity related to functional equivalence problem stems from the fact that different ways are possible according to which the source of the potential hazard can be defined.

When performing any form of comparative analysis it is essential that technological alternatives are compared either based on functional- or size equivalence criterion. It has to be acknowledged that risk assessment settings are not supposed to be the same when performing -

• comparisons between the individual landfills of different types vs. performing comparisons between the presumed individual landfills representing landfill types as groups, taking into account overwhelming internal diversity which exists among the landfills appertaining to each particular group
• comparisons between the landfills of equal capacities sited over the same footprint area vs. performing comparisons of landfills of functionally realistic capacities sited over the same footprint area

**Fig. 7:**
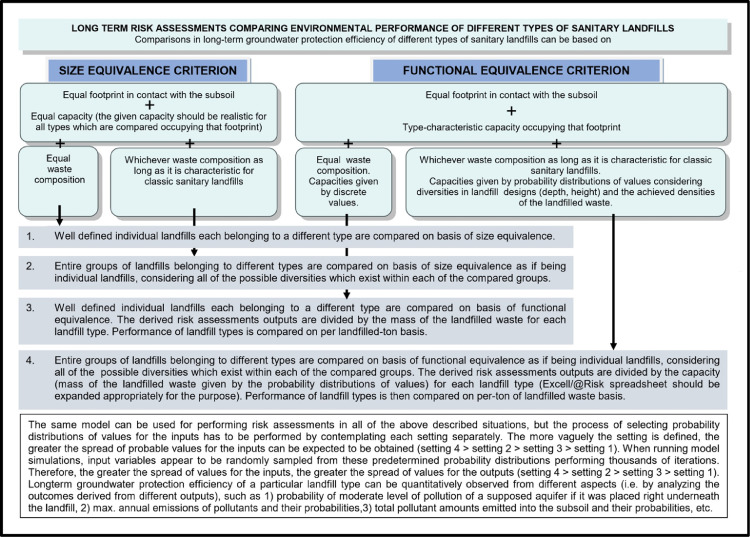
Demonstration of possible criterions to perform comparative risk assessments analysing differences between landfill types.

Therefore, different criteria could have been used to compare long-term groundwater protection efficiency of different types of sanitary landfills when referring to the research article [1]. Each of the four possibilities (which are graphically presented in Fig. 7) are contemplated below:

• The task of the study [1] was to compare environmental performance of four equally large sanitary landfills representing different landfill types in a wholistical way. *Size equivalence criterion #2* was therefore applied for the purpose. Characteristics of the pressumed landfill included whole range of possible heterogeneities and complexities which exist among the landfills appertaining to a particular landfill type, resulting in a rather wide spread of possible values for the inputs.
• The purpose of the analysis presented in the research article [1] could have been 'slightly' different, e.g., intended to compare long-term groundwater protection performance of four clearly defined individual landfills of four different types after their closure. In this case the common source of hazzard would have been defined as a singular 5 ha large sanitary landfill of 500.000 t capacity situated in a moderately humid environment which received waste with quite exactly known composition. Also, large quantity of historic monitoring data is assumed to exist for each of the four landfills (e.g., regarding quality and quantity of the primary leachate, composition and amount of the captured landfill gas, historic weather station data, etc.) as well as technical information (landfill design, mode of operation, etc.). In such a case *size equivalence criterion #1* (see Fig. 7) would be applied. However, even if the four landfills were so well defined, the problem still appears to be too complex to be solved deterministically. Inputs should still be attributed with probability distributions of possible values because of the uncertainty factor. By using the proposed method, both, #2 and #1 tasks can be assessed in the very same way, only that in the second case the selected distribution of possible values for particular uncertain inputs would be much less spread and the average values much more precise, dependent on the available amount of useful information.
• We could have been interested in comparing environmental performance of four sanitary landfills lying over the same footprint area as in the cases #1 and #2, which however did not receive the same amount of waste during their operational phases but rather received technically probable amounts of waste (therefore, functional differences which exist among different landfill types from the aspect of their probable landfill capacities over a particular landfill footprint area would be taken into account). The same model can be used, but there are some important differences. For example, modern landfills (usually designed as combined pit-and-mound facilities) generally accomodate two- three- four times more waste over the same footprint area than the low- density above- ground waste deposits. Defining the task in this way, we would be ultimately comparing long-term environmental performance of different landfill types on per-ton basis of the landfilled waste (i.e., *functional equivalence criterions #3 or #4* as presented in Fig. 7 would be applied). However, this does not mean that the related results solving problems #3 or #4 using the proposed methodology would be now shifted 2- 3 −4 times in favor of modern landfills comparing them to the results solving problems #1 or #2, although some shift in this direction is of course to be expected. It has to be taken in mind that risk assessment outcomes for a 5 ha large modern landfill with the capacity of e.g. 1.500.000 t would be worse than for a 5 ha large modern landfill with a capacity of 500.000 t Probability distributions of some of the inputs would not be the same in both of cases. Specifically, the parameter “rate of reference pollutant concentration decline” expressed as half-life period ‘T_0.5_’ would be shifted upwards (average ‘T_0.5_’ value would be larger) if the landfill capacity over the same footprint area was larger. In other words, higher, deeper, denser landfills stabilize more slowly on average than lower, shallower, less dense landfills, triggering greater environmental risks.

Many questions can arise out of this explanation. For example:

*Question 1:* Why is ‘T_0.5_’ the most important input which has to change when dealing with differences in landfill capacities over the same footprint area and not e.g. the input ‘C_0’_or the input 't_failure_^'^ (time needed for composite liner system to fail)?

*Question 2:* The overall pollution potential accumulated over the 5 ha footprint is ~3 times larger if the capacity of the modern landfill was 1.500.000 t instead of 500.000 t. Should not the calculated long term emissions into the subsoil or the acquired probabilities of aquifer contamination be approximately three times larger, too?

*Answer 1a:* ‘C_0’_is strongly related to factors such as (1) biodegradable matter content within the buried waste and (2) practice of sanitary covering used during the active waste disposal phases. These factors would remain basically the same if a 5 ha large modern landfill had the capacity of 500.000 t instead of 1.500.000 t. On the other hand, in the later case, the landfilled waste would be denser on average, initial aerobic phase shorter, liquid to solid ratio smaller and conditions in general more anaerobic. Therefore, the selected average ‘C_0’_value for a large capacity modern landfill would be generally greater than for a small capacity landfill of the same type streching over the same footprint area. However, average ‘C_0’_values for NH_4_—N are not likely to be larger than 1200 mg/L, since this value is already high for anaerobic landfills undergoing stable methanogenic phase (e.g., Table 2). Therefore, trying to find the right selection of average values for ‘C_0’_and ‘T_0.5_’ as a set is a better approach than trying to find the correct averages for ‘C_0’_and ‘T_0.5_’ separately.

*Answer 1b:* Eventual argument that major differences in probabilities for the parameter “time needed for composite bottom liner system to fail” have to be considered when comparing a 5 ha modern landfill which received 1.500.000 t of waste to the one which received only 500.000 t can not be reasonably substantiated. It's true, if landfill capacity was greater the overburden pressure would be larger and the amount of pollutants conveyed through the leachate drainage and conveyance systems would be ultimately larger, too. However, the probabilities for leachate drainage layer to be thicker and tension properties of the implemented geomembrane to be better would also be greater, cancelling the opposing factors out. Therefore, in general, the same probability distribution for the input 't_failure_’ would be used for both of settings.

*Answer 2:* Due to 3 times larger overall pollution potential, the potential for generating reference aqueous pollutant within the landfill appears to be ~3 times larger, too. However, most of this additional pollutant would be ultimately pumped out with the leachate. For modern landfills it is inherently expected leachate withdrawal and treatment systems are installed and function properly most of the time for the duration of at least 30 years after landfill closure. Therefore, this additional amount of pollutant would not have much effect on emissions into the subsoil. However, post-closure time until the time the landfill becomes stabilized would last longer, consequently, longer time would be on disposal for aquifer to become polluted. If ‘’mean value = 60 years’’ and ‘’st.dev. = 10 years’’ were used to characterize probability distribution of the input ‘T_0.5_’ reprezenting 1.500.000 t large dry-type modern landfill instead of ‘’mean = 40 years’’ and ‘’st.dev. = 7 years’’ reprezenting 500.000 t large landfill, the ultimate cumulative quantity of the reference pollutant released into the aquifer would increase to ~11.200 kg from ~7100 kg (both values given as 95 percentiles) by using spreadsheet model as presented in Fig. 5. Therefore, 3 times larger pollution potential accumulated at a particular site does not imply the related groundwater pollution potential to be 3 times larger, too.

Theoretically, we could have been interested in comparing environmental performance of four types of landfills (as presented in the Section ``*Types of sanitary landfills”*) lying over an 5 ha large footprint area (as in the cases #1, #2, #3 and #4), this time implementing size-equivalence criterion considering common landfill capacity of 1.500.000 t for all landfill types. However, such comparison would have been null and void, because neither above-ground dumpsites nor HPL's can exist occupying a footprint area of just 5 ha. It is up to a environmental engineer/scientist who performs comparative assessments to use the model appropriately, avoiding making functionally disequivalent comparisons.

## Modeling approach used to calculate contaminant transport through a HPL's compacted clay liner

Main points here are to explain

• why was advection the only transport mechanism considered to calculate migration of a reference pollutant through a CCL at the bottom of HPL-type of landfill as presented in Section ``*Approaches used to calculate leakings when referring to the companion research article”*.
• the reason for introducing FoS factor (i.e., Factor of Safety) into the HPL-related risk assessment mathematical model as presented in spreadsheet Fig. 4.

Once the CCL's hydraulic conductivity is lower than 1 × 10^−9^ m/s, molecular diffusion becomes more important pollutant transport mechanism than the simultaneously occuring advection mechanism [16]. Diffusion was not considered in the actual model which can be perceived as a deficiency. However, in contrast to conventional dry-type landfills, where concentration gradient through the clay liner situated underneath the (eventually) leaky geomembrane is large and lasts for centuries, this is not the case for HPL's. Concentration gradient in HPL's is low from the beginning and diminishes rapidly during the course of time after landfill closure because leachate quality improves fast through the years. After two decades, concentration gradient becomes too small to be considered as a relevant pollutant- transport- driving force any more even if high values for diffusion coefficient (*D* ≈ 10^−9^ m^2^/s) were considered for the calculation. Such condition develops decades before the pollutants manage to penetrate the CCL and break through on the other side of CCL.

As presented in the spreadsheet model (Fig. 4) fugitive flux of pollutants is acquired by multiplying time-dependent value ‘C_t_’ (reference pollutant concentration at the bottom of the landfill) with fugitive water flow through the clay liner ‘Q_t_’ driven by the hydraulic gradient which exists between the upper and lower CCL planes. Fugitive pollutant flux is therefore calculated at the upper CCL plane. However, leachate derived pollutants begin to be emitted into the subsoil only after the original, natural pore water was already largely pushed out of the CCL. ~80 years on average are needed for pollutants to penetrate the liner and break through on the other side (at the same time having in mind that a single simulation performing thousands of iterations consists of scenarios with calculated migration times as different as '35 years' and '120 years' post-closure (which is the consequence of the fact that the inputs 'hydraulic conductivity' and ‘CCL thickness' were attributed with probability density functions, not with discrete values).

Once the breakthrough occurs, the pollutant fluxes on the upper and lower CCL planes would be equal only if pollutant concentrations were equal on both sides. This would be theoretically the case only if long-term rate of decline in pollutant concentration within the primary leachate (expressed as half life period) would be the same as long-term rate of decline in concentration of pollutants which already infiltrated into the CCL. Processes involved in pollutant concentration decrease are however different within the two environments: biodegradation and washout of pollutants are the important processes going on within the landfill interior, while dispersion (mixing/dilution), retardation, irreversible sorption and biodegradation are the related simultaneously occuring important processes taking place within the CCL.

It is likely that long-term decrease in pollutant concentration is faster in CCL than in the landfill interior due to pulse-like initial input of pollutants into the CCL, allowing rapid dilution of the concentration plume (i.e., large bulk of pollutants penetrate the CCL during the first ten post- closure years). For modeling purposes, however (in order to be on the conservative side when performing risk assessments reffering to HPL as a low-cost landfill type), the product C_0_·EXP(-ln2/t_0.5_) · Q_t_ was multyplied with a “factor of safety” FoS = 100. The same result would be acquired by applying two times longer half-time for the parameter “reference pollutant decline in primary leachate” in the formula for calculating ‘C_t_’ (i.e., *t*_0.__5_ = 7 years instead of *t*_0.__5_ = 3.5 years).

Explanation from another perspective:

Concentration within the primary leachate decreases 100 times in 24 years when considering rate of decline employing half-time period t_0.__5_ = 3.5 years (as used in the model). By multiplying the formula with the “factor of safety” value of FoS = 100, the calculated pollutant flux *out of CCL* (through the bottom plane) appears to be equal to the pollutant flux *into the CCL* (through the upper plane) 24 years before.

## Model verification and validation

Risk derives from our inability to predict the future. Even though the outcome is uncertain, an objective risk can be described precisely based on theory or experiment. In contrast, describing the chance of a bottom liner to fail (defined as the chance that landfill- derived aqueous pollutants will be detected in the downstream monitoring well) is not clear and this represents a subjective risk. Given the same information, expert A might conclude there is a 80% chance some kind of failure will happen during the first 100 years after landfill closure whereas expert B might conclude the chance is only 50%. Neither of the two is necessarily wrong. Describing a subjective risk is open-ended in the sense that anyone's assessment could be always refined with new information, further study, using different approach or by giving weight to the opinion of others [11].

Also, deciding that something is risky requires personal judgment, even for objective risks. For example, many experts know or feel that dry-type landfills are generally safe facilities in the short-term but risky in the long-term if situated in hydrogeologically vulnerable environments. However, most people weight short-term risks much more critically than long-term risks. That's probably the main reason why dry-type modern landfills appear to be favored in many parts of the world which manifests itself in environmental regulations, too.

**Fig. 8:**
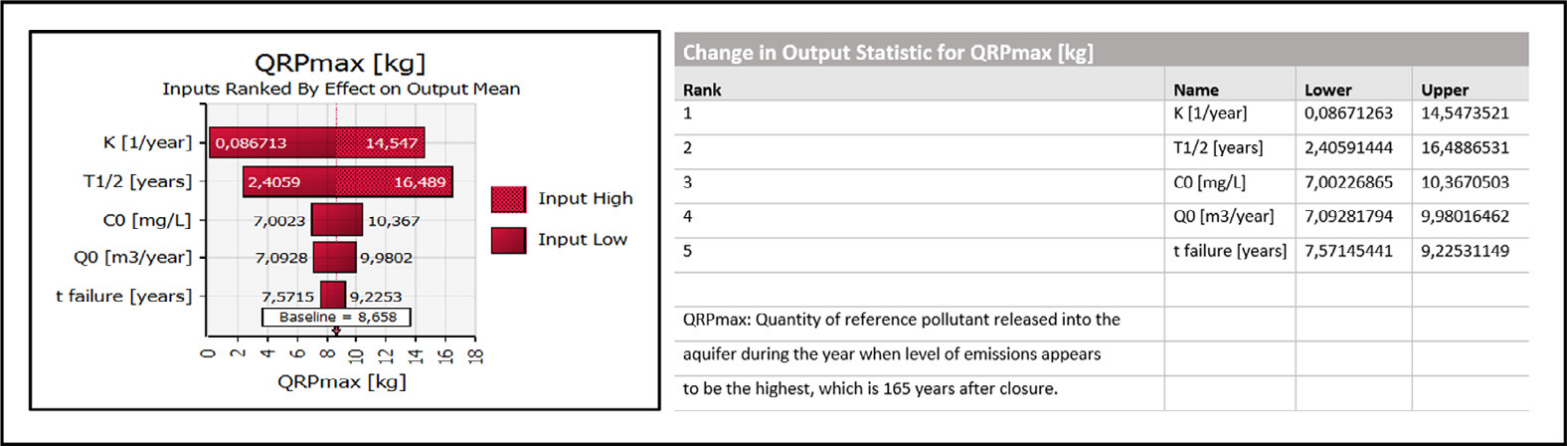
Example of an @Risk sensitivity analysis report.

So called “@Risk Output Reports” were included as a supplemental material to the companion data-description article [2]. Sensitivity analyses of some of the outputs (maximal annual emmision rates and maximal cumulative amount of the reference pollutant released into the environment) were an integral part of these reports. Input variables were ranked according to the effect they have on the outputs for each of the four landfill types. Sensitivities were presented in graphical and tabular forms evaluating the effects on the output averages if input values given by probability distributions were low, high or anything in between (see Fig. 8). As expected, the most critical inputs are the ones which are the least accessible and validable: “time needed for bottom liner to fail” (wet-type modern landfills), “rate of leakage increases after the system fails” (dry-type modern landfills) and “percentage of annual precipitation transformed into a leachate flow down to the aquifer” (above-ground dump sites).

Validation of a model assumes that measurements performed in the real world would confirm or deny the outcomes derived by modeling. However, a probabilistic model gives a distribution of possible outcomes as a result and gives some measure of how likely each outcome is to occur. Therefore, it is not possible to deterministicaly calibrate or validate a probabilistic model by performing measurements in the field; measured values will comply with the model as long as they fit within the range of probable values derived by modeling. Also, realistic landfills are not placed in the same environmental setting as were those which were evaluated in the model for purposes of performing comparative risk assessments [1]. Last but not the least, long term events will happen in the future, they can not be assessed in a real time.

Nevertheless, already available monitoring data acquired from the real world should agree well with the modeling results presented in the related research article [1] on an area unit (e.g., hectare) basis, because input parameters were also attributed with probability distributions of values taken from the real world - the applied model is robust enough. Assume a conventional dry type landfill which was closed 10 years ago somewhere in the USA. If the assumed landfill does not leak (i.e., zero emissions into the subsoil were detected 10 years after closure), compliance with the model would be excellent. According to the model, it is 85% probable NH_4_—N emissions would be zero, 90% probable to be smaller than 1.1 kg/(ha·year), 95% probable to be smaller than 1.9 kg/(ha·year), 99% probable to be smaller than 3 kg/(ha·year) and 100% probable NH4-N emissions would be smaller than 6.5 kg/(ha·year). Or, assume modern biorector landfill large 10 ha somewhere in developed world which was closed 5 years ago. If somehow fugitive emissions were detected and evaluated to be around 20 kg NH_4_—N/year, that would comply well with the given model, too (according to modeling results it is 90% probable emissions would be zero, 95% probable emissions would be smaller than 12 kg NH_4_—N, 99% probable they would be smaller than 35 kg and 100% probable they would be smaller than 62 kg).

First sector of the pilot HPL (Ajdovščina, Slovenia) which was receiving waste from the early 1980s was closed in 2005. Two boreholes located just 3 m apart were drilled into the landfill and screened at that time. Leachate samples from the bottom of the landfill are occassionally taken and analysed as well as samples representing extremely small quantities of interstitial water which exists at the interface between the natural clayey stratum and marly flysch lying 4.5 m underneath the landfill bottom. The interstitial water is still found to be uncontaminated with the leachate- derived NH_4_—N, which complies very well with the acquired modeling results.

Similarly to the outputs, probability distributions attributed to some inputs can not be deterministically validated, too. For example, technical life-times of landfill barrier systems in field-scale applications are largely unknown. Different models are used to assess long-term performance of bottom liner systems. Probability distribution provided by Pivato 2011 [12] appears to be exceptional since the related raw data were literally taken out of the real world. All of the myriad factors which could have been involved in landfill containment system failures were encompassed, which includes the impacts triggered by human factors, too. When looking from this standpoint, probability distribution used in the actual model was already validated in the field. Still, the degree to which the derived failure probability curve appears to be relevant for predicting events far into the future remains to be unknown.

## Declaration of Competing Interest

The Authors confirm that there are no conflicts of interest.

## References

[1] Madon I., Drev D., Likar J. (2019). Long-term assessments comparing environmental performance of different types of sanitary landfills. Waste Manag..

[2] Madon I., Drev D., Likar J. (2019). Long-term groundwater protection efficiency of different types of sanitary landfills: data description. Data Brief.

[3] Xie H.J., Chen Y.M., Lou Z.H. (2010). An analytical solution to contaminant transport through composite liners with geomembrane defects. Sci. China Technol. Sci..

[4] Environment Agency (2004). LandSim 2.5 – Groundwater Risk Assessment Tool for Landfill Design.

[5] Turner A.D., Beaven P.R., Woodman D.N. (2017). Evaluating landfill aftercare strategies: a life cycle assessment approach. Waste Manag..

[6] Laner D. (2011). Understanding and Evaluating Long-Term Environmental Risks from Landfills. https://www.wien.gv.at/umweltschutz/nachhaltigkeit/pdf/laner.pdf.

[7] Council of the EU (1999). Landfill directive. Off. J. Eur. Commun. L.

[8] Hjelmar O., Andersen L., Hansen J.B. (2000). Leachate Emissions From Landfills.

[9] Madon I. (2015). A case study of an holistic approach to leachate and storm-water management developed at a municipal landfill site. WIT Trans. Ecol. Environ. Water Resour. Manag. VIII.

[10] Madon I. (2016). Development of a sustainable msw landfill as an intrinsic part of a low-priced, integrated waste management facility. WIT Trans. Ecol. Environ. 202. Waste Manag..

[11] Palisade Corporation (2016). @Risk Analysis and Simulation, Add-in for Microsoft Excell. http://www.palisade.com.

[12] Pivato A., Failure Landfill Liner (2011). An open question for landfill risk analysis. J. Environ. Prot..

[13] DEFRA U.K. (2011). Green Leaves III – Guidelines for Environmental Risk Assessment and Management. https://assets.publishing.service.gov.uk/government/uploads/system/uploads/attachment_data/file/69449.

[14] US EPA (2002). Assessment and Recommendations for Improving the Performance of Waste Containment Systems.

[15] Rowe R.K. (2012). Short and long-term leakage through composite liners. Can. Geotech. J..

[16] Rowe R.K. (2013). The role of diffusion in environmental geotechnics. Proceedings of the 18th International Conference on Soil Mechanics and Geotechnical Engineering.

[17] Boerboom A.A.M., Foppen E., van Leeuwen O., Christensen T.H., Cossu R., Stegmann R. (2003). Risk assessment methodology for aftercare of landfills based on probabilistic approach. Proceedings of Sardinia 2003, 9th International Waste Management and Landfill Symposium.

[18] Rodic-Wiersma L.J., Goosens L.H.J., Christensen T.H., Cossu R., Stegmann R. (2001). Assessment of landfill technology failure. Proceedings of Sardinia 2001, 8th International Waste Management and Landfill Symposium.

[19] Giroud J.P., Khire M.V., Siderman K.L. (1997). Liquid migration through defects in geomembrane overlain and underlain by permeable media. Geosynth. Int..

[20] Beck A. (2014). How much does my landfill leak?. Waste Advant. Mag..

[21] Jingjing F. (2014). Leakage performance of the gm+ccl liner system for the msw landfill. Sci.c World J..

[22] Ramke G., Telekes G., Imre E., Witt K.J., Ramke G. (2009). Leachate collection systems. Proceedings of the 1st Middle European Conference on Landfill Technology.

[23] Bouchez T., Munoz M.L., Vessigaud S., Bordier C., Aran C., Duquennoi C. (2003). Clogging of msw landfill leachate collection systems: prediction methods and in situ diagnosis. Proceedings of Sardinia 2003, 9th International Waste Management and Landfill Symposium.

[24] Rowe R.K., Vangulck J., Millard S. (2002). Biologically induced clogging of a granular medium permeated with synthetic leachate. Can. J. Environ. Eng. Sci..

[25] Rowe R.K., Islam M.Z. (2009). Impact of landfill liner time-temperature history on the service life of hdpe geomembranes. Waste Manag..

[26] Geoservices Inc., Background Document on Bottom Liner Performance in Double-Lined Landfills and Surface Impoundments, EPA 1530-SW-87-013, US EPA, 1987.

[27] Moo-Young H., Johnson B., Carson D., Lew C., Liu S., Hancocks K. (2004). Characterization of infiltration rates from landfills: supporting groundwater modeling efforts. Environ. Monit. Assess..

[28] Y.G. Hsuan, H.F. Schroeder, K. Rowe, W. Müller, J. Greenwood, D. Cazzuffi, R.M. Koerner, Long Term Performance and Lifetime Prediction of Geosynthetics, EuroGeo4 Keynote Paper, 2009.

[29] Council of the EU (2003). Establishing criteria and procedures for the acceptance of waste at landfills pursuant to Article 16 and Annex II to directive 1999/31/EC. Off. J. Eur. Commun..

[30] Kjeldsen P., Barlaz A.M., Rooker P.A., Baun A., Ledin A., Christensen H.T. (2002). Present and long-term composition of msw landfill leachate: a review. Crit. Rev. Env. Sci. Technol..

[31] Lou Z., Dong B., Chai X., Song Y., Zhao Y., Zhu N. (2009). Characterization of refuse landfill leachates of three different stages in landfill stabilization process. J. Environ. Sci..

[32] Stegmann R., Ritzkowsky M. (2007). Landfill Aeration.

[33] Reinhart D.R., Townsend T.G. (1998). Landfill Bioreactor Design and Operation.

[34] Bolyard S.C., Reinhart D.R. (2016). Application of landfill treatment approaches for stabilization of municipal solid waste. Waste Manag..

[35] Price G.A., Barlaz M.A., Hater G.R. (2003). Nitrogen management in bioreactor landfills. Waste Manag..

[36] Ahmadifar M., Sartaj M., Abdallah M. (2015). Investigating the performance of aerobic, semi-aerobic and anaerobic bioreactor landfills for msw management in developing countries. J. Mater.Cycles Waste Manag..

[37] Qu X., He P., Shao L., Lee D. (2008). Heavy metals mobility in full-scale bioreactor landfill: initial stage. Chemosphere.

[38] Kumar D., Allapat B.J. (2005). Evaluating leachate contamination potential of landfill sites using leachate pollution index. Clean Technol. Environ. Policy.

[39] Fellner J., Dőberl G., Allgaier G., Brunner P.H. (2009). Comparing field investigations with laboratory models to predict landfill leachate emissions. Waste Manag..

[40] Cossu R., Lavagnolo M.C., Raga R., Stegmann R., Ritzkowsky M. (2007). In-situ stabilization of old landfills: lab scale and field tests. Landfill Aeration.

[41] Matsufuji Y. (2004). A road to semi-aerobic landfill. Proceedings of the Third Intercontinental Landfill Research Symposium.

[42] Shimaoka T., Matsufuji Y., Hanashima M. (2000). Mechanism of self-stabilization of semi-aerobic landfill. Proceedings of the 5th Annual Landfill Symposium.

[43] Grossule V., Lavagnolo M.C. (2017). Innovative semi-aerobic landfill management in tropical countries. Proceedings of Sardinia 2017, 16th International Waste Management and Landfill Symposium.

[44] Noopharit S., Chiemchaisri C., Wangyiao K., Rowprayoon S., Endo K., Yamada M. (2014). Comparison of solid waste stabilization and methane emission from anaerobic and semi-aerobic landfills operated in tropical condition. Environ. Eng. Res..

[45] F.G. Pohland, S.R. Harper, Critical Review and Summary of Leachate and Gas Production from Landfills, EPA/600/2-86/073, PB86-240181, 1986.

[46] S. Cestaro, D. Rossetti, R. Cossu, Full-scale application of Aerobic in Situ Stabilization of an Old Landfill in North Italy, 2006. https://www.researchgate.net/scientific-contributions/2058438734_S_Cestaro

[47] Barlaz M.A., Rooker A.P., Kjeldsen P., Gabr M.A., Borden R.C. (2002). Critical evaluation of factors required to terminate the postclosure monitoring period at solid waste landfills. Environ. Sci. Technol..

[48] Ehrig H.J. (1983). Quality and quantity of sanitary landfill leachate. Waste Manag. Res..

[49] Kjeldsen P., Christophersen M. (2001). Composition of leachate from old landfills in Denmark. Waste Manag. Res..

[50] Sarsby R.W. (2000). Environmental Geotechnics.

[51] Aziz A.H., Hosseini M. (2012). Penang experience in solid waste disposal by semi-aerobic sanitary landfill. Proceedings of Brunei International Conference on Engineering and Technology.

[52] U.S. EPA (1995). Compilation of Air Pollutant Emission factors: Municipal solid Waste landfills, Office of Air Quality Planning and Standards.

[53] Berge N.D., Reinhart D.R., Dietz J., Townsend T., Stegmann R., Ritzkowsky M. (2007). In-situ ammonia removal in bioreactor landfill leachate. Landfill Aeration.

[54] Gross B.A., Bonaparte R., Giroud J.P., Bonaparte R., Daniel D.E., Koerner R.M. (2002). Waste containment systems: problems and lessons learned, appendix F. Assessment and Recommendations for Optimal Performance of Waste Containment Systems.

[55] Sullivan P.S., Stege G.A. (2000). An evaluation of air and greenhouse gas emissions and methane recovery potential from bioreactor landfills. MSW Manag..

[56] Tolaymat T.M., Green R.B., Hater G.R., Barlaz M.A., Black P., Bronston D., Powell J. (2010). Evaluation of landfill gas decay constant for municipal solid waste landfills operated as bioreactors. J. Air Waste Manag. Assoc..

